# Selection of Image Texture Analysis and Color Model in the Advanced Image Processing of Thermal Images of Horses following Exercise

**DOI:** 10.3390/ani12040444

**Published:** 2022-02-12

**Authors:** Małgorzata Domino, Marta Borowska, Natalia Kozłowska, Anna Trojakowska, Łukasz Zdrojkowski, Tomasz Jasiński, Graham Smyth, Małgorzata Maśko

**Affiliations:** 1 Department of Large Animal Diseases and Clinic, Institute of Veterinary Medicine, Warsaw University of Life Sciences (WULS–SGGW), 02-787 Warsaw, Poland; malgorzata_domino@sggw.edu.pl (M.D.); natalia_kozlowska@sggw.edu.pl (N.K.); tomasz_jasinski@sggw.edu.pl (T.J.); 2 Institute of Biomedical Engineering, Faculty of Mechanical Engineering, Białystok University of Technology, 15-351 Bialystok, Poland; m.borowska@pb.edu.pl; 3 The Scientific Society of Veterinary Medicine Students, Warsaw University of Life Sciences (WULS–SGGW), 02-787 Warsaw, Poland; an.trojakowska@gmail.com; 4 Menzies Health Institute Queensland, Griffith University School of Medicine, Southport, QLD 4222, Australia; grahamcsmyth@gmail.com; 5 Department of Animal Breeding, Institute of Animal Science, Warsaw University of Life Sciences (WULS–SGGW), 02-787 Warsaw, Poland

**Keywords:** effort, infrared thermography, image texture, noninvasive, screening measures

## Abstract

**Simple Summary:**

Detecting horse state after exercise is critical for maximizing athletic performance. The horse’s response to fatigue includes exercise termination or exercise continuation at a lower intensity, which significantly limit the results achieved in races and equestrian competition. As conventional methods of detecting and quantifying exercise effort have shown some limitations, infrared thermography was proposed as a method of contactless detection of exercise effect. The promising correlation between body surface temperature and exercise-dependent blood biomarkers has been demonstrated. As the application of conventional thermography is limited by low specificity, advanced thermal image analysis was proposed here to visualize the link between blood biomarkers and texture of thermal images. Twelve horses underwent standardized exercise tests for six consecutive days, and both thermal images and blood samples were collected before and after each test. The images were analyzed using four color models (RGB, red-green-blue; YUV, brightness-UV-components; YIQ, brightness-IQ-components; HSB, hue-saturation-brightness) and eight texture-features approaches, including 88 features in total. In contrast to conventional temperature measures, as many as twelve texture features in two color models (RGB, YIQ) were linked with blood biomarker levels as part of the horse’s response to exercise.

**Abstract:**

As the detection of horse state after exercise is constantly developing, a link between blood biomarkers and infrared thermography (IRT) was investigated using advanced image texture analysis. The aim of the study was to determine which combinations of RGB (red-green-blue), YUI (brightness-UV-components), YIQ (brightness-IQ-components), and HSB (hue-saturation-brightness) color models, components, and texture features are related to the blood biomarkers of exercise effect. Twelve Polish warmblood horses underwent standardized exercise tests for six consecutive days. Both thermal images and blood samples were collected before and after each test. All 144 obtained IRT images were analyzed independently for 12 color components in four color models using eight texture-feature approaches, including 88 features. The similarity between blood biomarker levels and texture features was determined using linear regression models. In the horses’ thoracolumbar region, 12 texture features (nine in RGB, one in YIQ, and two in HSB) were related to blood biomarkers. Variance, sum of squares, and sum of variance in the RGB were highly repeatable between image processing protocols. The combination of two approaches of image texture (histogram statistics and gray-level co-occurrence matrix) and two color models (RGB, YIQ), should be considered in the application of digital image processing of equine IRT.

## 1. Introduction

Detecting animal fatigue after exercise is critical to ensure maximal performance in athletes [1]. Horses are considered the most utilized athletes within the animal kingdom. Thus, huge investments of time and money are made to increase their exercise capacity and improve their overall performance during training [2,3,4]. Exercise-induced fatigue occurs after repeated exercise with insufficient recovery [5,6] and/or prolonged [7,8] or high-intensity exercise [4,9]. Exercise-induced fatigue results in temporary loss of strength and energy, leading to decreased capacity for physical activity and a decline in the execution of horses’ athletic skills [2,10]. A horse’s response to fatigue can include exercise termination or exercise continuation at a lower intensity [4,11], both of which significantly limit the results achieved in races [4,9] and the equestrian Olympic games [12,13,14]. Hence, exercise should include evaluation of horse state in order to avoid fatigue [2,3,4]. Performance decrease is associated not only with a decrease in energy source in muscles but also hormonal changes causing mental stress and welfare disturbance [12,15].

Conventional methods of detecting and quantifying exercise effort and intensity include measuring blood biomarkers [4,16], heart rate (HR), heart rate variability (HRV) [5,17], and other non-invasive techniques [18,19]. Equine blood biomarkers are categorized as follows: ATP metabolism biomarkers, such as lactate concentration (LAC); muscle damage biomarkers, such as creatine phosphokinase (CPK) and aspartate aminotransferase (AST) activity; and inflammatory biomarkers, such as white blood cell count (WBC) [4,16]. Non-invasive measures typically involve surface electromyography (EMG) [18]; infrared thermography (IRT) [15,19]; telemetric monitoring of speed, gait, and HR [9,20]; as well as kinematic monitoring of gait [20]. Although these methods are used in equine veterinary practice, advanced applications as a non-invasive, contactless method of detecting exercise effect after repeated exercise are limited. Biomarkers require blood sampling, and the results are not instantaneously available, as processing and analysis are required [4,6,7]. HR is highly susceptible to the effect of a horse’s emotional response, especially stress [21,22]. Equine EMG, recording site-specific stimulation via electrodes placed on the muscle [18], requires application of surface electrodes, which is challenging during movement [20], or the insertion of a hypodermic needle, which makes EMG invasive and causes discomfort [23]. Both surface and needle/fine-wire EMG require specialist equipment and training in how to collect and/or analyze recorded data [18,20]. Despite equine IRT being susceptible to environmental effects [19,24,25], it appears to be the most accessible method, as it allows for contactless quantification of radiant energy emitted by the body surface, which is proportional to the horse’s effort intensity [26,27]. Promising correlations have been demonstrated between body surface temperature and exercise-dependent blood biomarkers after a single training session [15,27]. However, the long-term relations after repeated exercise have hitherto been unstudied.

Images directly acquired from IRT have limitations, especially considering their specificity. Images without processing do not differentiate between influences that produce different surface temperatures [28]. Fortunately, in recent times, digital image processing has been developed for more comprehensive IRT image analysis. Such image processing provides quantified, objective data considered to be more informative than conventional temperature measures in the fields of material mechanics [29,30,31] and human medicine [32,33,34,35,36,37]. Digital image processing has been applied successfully in the detection of damage to composite materials [29], aerospace structures [30], and building elements [31], thus increasing the efficiency of non-destructive material testing. Similarly, increased efficiency has been reported in the detection of diabetes [32], skin cancer [33], and breast tumors [34]. Digital image analysis of thermal images is an innovative and favorable approach in many disciplines. However, to the best of our knowledge, to date, our preliminary work has been the only application in equine medicine [38,39]. Recent use of digital image processing in equine research is based on gray-level matrices (GLM), which has been used to detect the influence of a rider’s body weight on the thermography of the thoracolumbar region [39]. Technological developments allow for the evaluation of relationships between recognized biomarkers of exertion and IRT advanced image texture analysis. This would provide a contactless method for the detection of exertion levels in horses. 

In the current study, eight digital image-processing approaches in four color models were applied to evaluate exercise effort and intensity after repeated exercise. Thermal images of horses’ thoracolumbar region were reproduced in red/green/blue (RGB), brightness/U component/V component (YUV), brightness/I component/Q component (YIQ), and hue/saturation/brightness (HSB) color models to carry out histogram statistics (HS), gradient map (GM), autoregressive model (AM), Gabor transform (GT), histogram of oriented gradients (HOG), and gray-level matrices (GLM) approaches. GLM is represented here by three detailed approaches: gray-level run-length matrix (GRLM), symmetric gray-level co-occurrence matrix (GLCM), and asymmetric gray-level co-occurrence matrix (GLCH). GLM describes a group of texture operators that map image function, image complexity, and statistics of pixels distribution [35]. The GLM approach has been reported to provide the best description of image texture [35,36]. It has previously been used for extraction of features of medical images, e.g., ultrasound images [37], radiographic images [40,41], magnetic resonance images [36,42], and thermal images [39]. 

In this study, we aimed to determine color-model component combinations and image texture features that correlate with conventional measures (bloodborne biomarkers) of exercise-induced changes over multiple exercise trials. The identified features are to be used in future research investigating the application of digital IRT image processing in equine veterinary medicine.

## 2. Materials and Methods

### 2.1. Animals

Twelve horses (*n* = 12) (mean ± SD: age 9.3 ± 1.8 years, body weight 566.7 ± 13.7 kg, height at the withers 160.3 ± 3.9 cm; 6 geldings, 6 mares) stabled at the Didactic Stable of the Horse Breeding Division at Warsaw University of Life Sciences were used for the study. The horses represented three Polish warmblood breeds: Polish Halfbred horse (*n* = 7), Wielkopolska (*n* = 3), and Malopolska (*n* = 2) breeds. All horses were housed in individual stalls with the same management. Horses were fed an individually calculated ration of hay, oats, and concentrate according to their nutritional requirements, distributed over three feedings per day. A mineral salt block and freshwater were constantly available. All horses were physically fit as, general riding-school horses taking part in leisure riding for up to 2 h per day, 6 days a week. None of the horses was on any medication during sampling and for the 2 weeks prior to sampling. To exclude unhealthy horses from the study, clinical examinations were conducted before sampling. Basic clinical examinations included measurement of heart rate, respiratory rate, capillary refill time, and rectal temperature, as well as inspection of mucous membranes and lymph nodes. Examinations were conducted according to international veterinary standards [43]. Detailed examination of the musculoskeletal system was performed following guidelines for lameness evaluation of the athletic horse [44]. Only horses showing no clinical signs and those of similar athletic ability were included in the research. None of the horses was excluded. This study was approved by the II Local Ethical Committee on Animal Testing in Warsaw on behalf of the National Ethical Committees on Animal Testing (No WAW2/034/2018, day 27 April 2018). 

### 2.2. Study Design

Data were collected directly after exercise to measure its effect following a protocol adapted from Munsters et al. [45] whereby horses were subjected to standardized exercise tests (SETs) repeated daily for six days as six repetitions of exercise (REs). During each RE, heart rate (HR), velocity (v), and blood lactate concentration (LA) were measured to achieve a repeatable level of effort. During REs, horses were equipped with an HR monitor (Polar-RS800, Polar Electro Oy, Kempele, Finland) that simultaneously recorded HR (beats/min) and speed (m/s). A first electrode was placed under the girth behind the left elbow, and the second was placed cranially to the withers and saddle. 

Each RE comprised a warm-up walk (5 min; up to 1.5 m/s), trot (10 min; up to 4.0 m/s), and four incremental exercise steps in canter (2.5 min, 5 min, 7.5 min, and 10 min; up to 6.0 m/s). After each exercise step, horses were slowed down and walked for 5 min. Within 1 min of the walk, horses were briefly stopped, and blood samples were collected from the jugular vein at rest to confirm the level of effort during RE. Plasma LA (mmol/L) was measured immediately with a portable handheld lactate measurement device (Accusport^®^, Roche Diagnostics, Basel, Switzerland). As lactate concentrations <1.0 mmol/L were under the detection limit, these values were all set at 1.0 mmol/L. The SET was deemed completed when HR reached 140 beats/min and the blood lactate concentration was at or above 4 mmol/L. After completion of each RE, horses did a rope-led walking cool-down until complete recovery was achieved at a HR level of 40–50 beats/min. 

Data collection included blood sampling (BS) and IRT imaging (II). Both data collections were jointly marked with the BS abbreviation for standardization of research transparency. BSs were repeated twice during each RE; the first sample was collected at rest before the RE (BS 0), and a second sample was taken immediately after completion of the RE and before the cool-down walk (BS 1). After BS 0, horses were saddled, and the RE was started. Before BS 1, horses were unsaddled, and the second thermographic image was taken. Before starting the research, the saddles were fitted to each horse following the protocol of Greve and Dyson [46]. The panels of the saddle, the type of flocking, and the balance of the saddle were determined. Only saddles considered to fit properly were approved for use in the research. In an effort to limit bias, only two female riders (body weight: 55.5 ± 0.7 kg; height: 163.0 ± 1.4 cm) with 6 years of riding experience in the upper-intermediate rider-training level participated in the study. Riders were members of the Animal Sciences Students Riding Association.

All REs and BSs were performed in an indoor riding hall with constant environmental conditions and protection from solar radiation and wind. Ambient temperature (°C) and relative humidity (RH; %) were continuously measured and, during Res, maintained at 20.1 ± 0.9 °C and 50.5 ± 2.8%, respectively. The riding surface was composed of silica sand and fiber fleece and was regularly watered by an automatic floor-watering facility to maintain the right level of moisture. The hall was directly connected with the horses’ stable; therefore, horses could participate in the research without having contact with the outside environment.

### 2.3. Blood Sampling and Biomarker Measurement 

Blood samples were acquired by jugular venipuncture using a BD Vacutainer^®^ system into K2-EDTA tubes for hematological tests and dry tubes for biochemical analyses (Plymouth, UK). K-2 EDTA blood samples were kept at +4 °C and examined within 5 h in an automated analyzer calibrated for equine species (ABC Vet, Horiba ABX). The following eight hematological parameters were considered: white blood cell count (WBC, ×10^9^/L), red blood cell count (RBC, ×10^12^/L), hemoglobin concentration (HGB, mmol/L), hematocrit (HCT, %), mean corpuscular volume (MCV, fL), mean corpuscular hemoglobin (MCH, gL), and mean corpuscular hemoglobin concentration (MCHC, mmol/L). The dry tubes were centrifuged (2000× *g*, 5 min), and serum free from any apparent hemolysis was aspirated and frozen at −20 °C for further analyses. After all BS collection, serum samples were defrosted and examined in an automated clinical biochemistry analyzer (Miura One, ISE. S.r.l., Rome, Italy) using Pointe Scientific (5449 Research Dr, Canton, MI, USA) reagents, standards, calibrators, and controls. The following five biochemical parameters were considered: blood lactate concentration (LAC, mmol/L), total serum protein concentration (TSP, g/L), creatine phosphokinase activity (CPK, U/L), alanine aminotransferase activity (ALT, U/L), and aspartate aminotransferase activity (AST, U/L).

### 2.4. IRT Data Collection and Analysis

**Figure 1:**
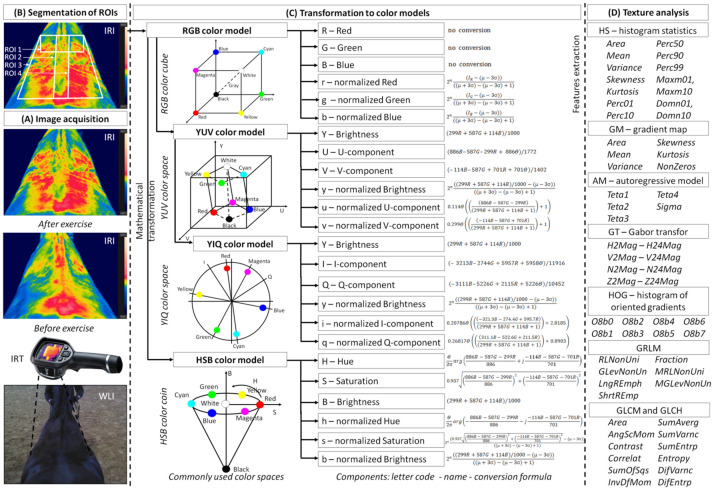
Thermal image processing steps for the analysis of image texture. The first step—image acquisition (**A**); the second step—segmentation of ROIs (**B**); the third step—mathematical transformation to color models (**C**); the fourth step—feature extraction during analysis of image texture (**D**). ROI—region of interest; WLI—white-light image; IRT—infrared thermography; IRI—infrared image; RGB—red/green/blue color model; YUV—brightness/U component/V component color model; YIQ—brightness/I component/Q component color model; HSB—hue/saturation/brightness color model; GRLM—gray-level run-length matrix; GLCM—symmetric gray-level co-occurrence matrix; GLCH—asymmetric gray-level co-occurrence matrix.

The imaged area of the thoracolumbar region was brushed 30 min before imaging so as to remove dirt and mud. The horses were then led to an enclosed, indoor riding hall to acclimatize to imaging conditions. Images were taken using a non-contact thermographic camera (FLIR Therma CAM E25, FLIR Systems Brasil, Sorocaba, Brazil; emissivity (e) 0.99; temperature range between 10.0 and 50.0 °C) (Figure 1A). The camera was placed approximately 1.2 m above the imaging area, directly above the L5 dorsal spinous process. All thermographic images were obtained by the same researcher (M.M.). The thermal image processing steps for conventional analysis of IRT measures included image acquisition, segmentation of regions of interest (ROIs), and extraction of the maximal temperature (Tmax) and average temperature (Taver) from given ROIs. Professional software (FLIR Tools Professional, FLIR Systems Brasil, Sorocaba, Brazil) was used for evaluations. Tmax represented the values of the highest temperatures recorded in consecutive ROIs, whereas Taver reported the value of the mean temperature calculated for the entire ROI area. The imaged thoracolumbar region was segmented into four ROIs representing the withers area (ROI 1), the thoracic spine area (ROI 2), the left area of back muscles (ROI 3), and the right area of back muscles (ROI 4), as displayed in Figure 1B.

### 2.5. Image Texture Analysis

The steps for thermal image processing of the image texture analysis include image acquisition, segmentation of ROIs, transformation to color models, and extraction of features using eight analytical approaches (Figure 1). The first and second steps were the same for the conventional analysis of IRT measures and image texture analysis. The third and fourth steps were those of digital image processing, leading to quantification of data from IRT images. The image texture features were extracted using eight computer-aided approaches in each of four annotated ROIs by QMazda Software [47,48].

#### 2.5.1. Color Models

**Figure 2:**
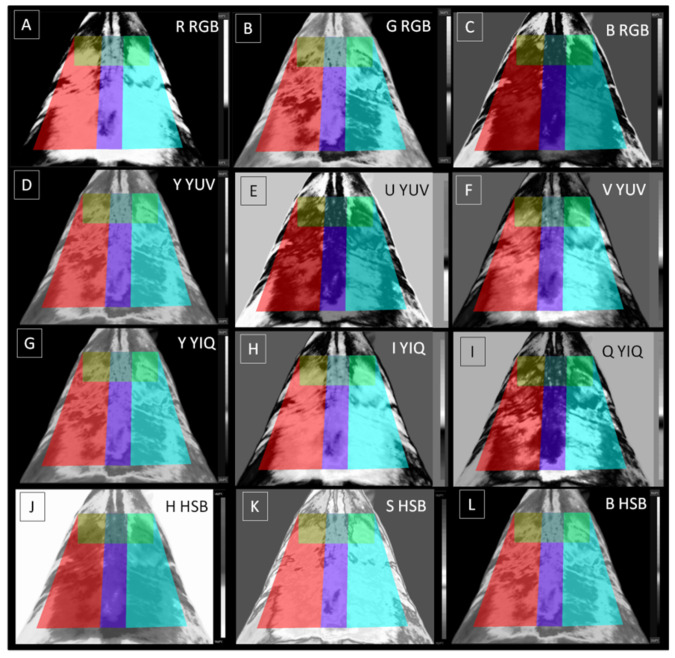
Sample imaging of a horse’s thoracolumbar region after conversion to red channel (**A**), green channel (**B**), and blue channel (**C**) in the RGB color model; brightness channel (**D**), U-component channel (**E**), and V-component channel (**F**) in the YUV color model; brightness channel (**G**), I-component channel (**H**), and Q-component channel (**I**) in the YIQ color model; as well as hue channel (**J**), saturation channel (**K**), and brightness channel (**L**) in the HSB color model. RGB—red/green/blue color model; YUV—brightness/U component/V component color model; YIQ—brightness/I component/Q component color model; HSB—hue/saturation/brightness color model.

The extraction of image texture features using QMazda Software requires a grayscale image as input. IRT images are colorful, and temperature is determined by color changes. Hence, the color analysis included the analysis of IRT image red (R), green (G) and blue (B) components in the RGB color model, as well as analysis of IRT image brightness (Y), U component (U), V component (V), I component (I), Q component (Q), hue (H), saturation (S), and brightness (B) components after transformation to YUV, YIQ, and HSB color models, respectively. IRT image features were calculated independently for individual components. In the RGB color cube, each of the RGB components depends proportionally on the intensity of the illumination and thus on the distance between the imaged object and the source of illumination. In other color spaces, chrominance components are separated, carrying information about the color (independent of brightness) and luminance (determining the brightness level). Spaces with separate luminance and chrominance components were obtained by transforming RGB spaces following the formulas in Figure 1C. Q-Mazda Software was used to extract the features from the selected component of each color model after transformation to grayscale by conversion to R, G, B, Y, U, V, I, Q, H, and S channels (Figure 2).

#### 2.5.2. Normalization

Brightness and contrast of IRT images were normalized, as feature variation may have an undesirable effect on the values of the extracted feature. Procedures for normalizing the gray level of the image were performed using Q-Mazda Software. Normalization procedures were carried out as follows: no normalization, using the mean, μ, and standard deviation, σ, of the gray levels (the range of computation is 〈μ−3σ; μ+3σ〉), using the minimum (*min*) and maximum (*max*) gray levels in the region of interest (the range of computation is 〈min; max〉), using the histogram percentiles (the range of computation is 〈p1; p99〉). Normalization was conducted according to the formula:

(1) $$I_{OUT}=2^{n}\frac{I_{IN}-MIN}{MAX-MIN+1}$$

where *I_IN_* is an original image, <*MIN*; *MAX*> defines a new range due to the chosen standardization procedure, and *n* defines the number of bits per pixel. The channels normalized in Q-Mazda Software were annotated using lowercase letters: r, g, b, y, u, v, i, q, h, and s. These were used to extract the features from the selected component independent of un-normalized channels.

#### 2.5.3. Image Texture Analysis

The extraction of image texture features was conducted by QMazda Software using eight approaches (Figure 1D), as follows:

- Histogram statistics (HS) use first-order histogram analysis, i.e., a function determined in the domain of image brightness without taking into account the spatial dependence of the brightness distribution [49]. The fourteen features obtained from histogram analysis are: area (HistArea), mean (HistMean), variance (HistVariance), skewness coefficient (HistSkewness), kurtosis (HistKurtosis), percentiles (HistPerc01, HistPerc10, HistPerc50, HistPerc90, and HistPerc99), dominants (HistDomn01 and HistDomn10), and maximum of moments (HistMaxm01 and HistMaxm10).
- Gradient map (GM) evaluates the spatial relationships present in the image by its transformation, i.e., by calculating the absolute value of the brightness gradient at each point in the image [49]. In the resulting image, local brightness variations between homogeneous areas of the original image are visible. The gradient is calculated as the root of the second degree of the sum of the squares of light derivatives in perpendicular directions, e.g., horizontal and vertical. Based on the histogram of the absolute value of the gradient, six statistical features are calculated: absolute gradient area (GradArea), absolute gradient mean (GradMean), absolute gradient variance (GradVariance), absolute gradient skewness (GradSkewness), absolute gradient kurtosis (GradKurtosis), and percentage of pixels with nonzero gradient (GradNonZeros).
- Autoregressive model (AM) assumes interaction between image pixels. The image is transmitted in lines from top to bottom, and each line is sent pixel by pixel from left to right. That pixel brightness can be predicted based on the brightness of previously transmitted pixels [50]. The algorithm returns five features relating the brightness of a pixel to its neighbors from the left (Teta1), top left (Teta2), top (Teta3), and top right (Teta4), as well as the minimum mean square error between the predicted and actual brightness (sigma).
- Gabor transform (GT) is image transformation consisting of local signal decomposition into frequency components [51]. Frequency components are calculated by convolution of the image with a Gaussian kernel. The features obtained from GT are defined using a combination of frequency, orientation (horizontal, vertical, 22.5°, 45°, 67.5°, 112.5°, 135°, and 157.5°), standard deviation of the Gaussian envelope (σ), and magnitude. The algorithm returns twenty-four features: Gab4H2Mag, Gab4V2Mag, Gab4N2Mag, Gab4Z2Mag, Gab6H3Mag, Gab6V3Mag, Gab6N3Mag, Gab6Z3Mag, Gab8H4Mag, Gab8V4Mag, Gab8N4Mag, Gab8Z4Mag, Gab12H6Mag, Gab12V6Mag, Gab12N6Mag, Gab12Z6Mag, Gab16H8Mag, Gab16V8Mag, Gab16N8Mag, Gab16Z8Mag, Gab24H12Mag, Gab24V12Mag, Gab24N12Mag, and Gab24Z12Mag.
- Histogram of oriented gradients (HOG) counts occurrences of gradient orientations. HOG is constructed using the gradient magnitude and orientation around the image pixel [52]. The algorithm returns eight features identified by the number of angular bins: 4b (HogO8b0, HogO8b1), 8b (HogO8b2, HogO8b3), 16b (HogO8b4, HogO8b5), or 32b (HogO8b6, HogO8b7).
- Gray-Level Run-Length Matrix (GRLM) gives the information about the number of pixel strings with the same brightness and specified length based on the pixel string length matrix [53]. The GRLM is computed for four different directions of the horizontal, vertical, 45°, and 135° pixel strings. The following basic seven features are calculated from this matrix: run-length nonuniformity (RLNonUni), gray-level non-uniformity (GLevNonUn), moment of long string emphasis (LngREmph), reverse moment of short string emphasis (ShrtREmp), fraction of image in runs (Fraction), run-length nonuniformity moment (MRLNonUni), and gray-level non-uniformity moment (MGLevNonUn).
- Gray-level co-occurrence matrix (GLCM) uses the second-order histogram of the image brightness distribution to determine the mutual spatial relationship between pairs of image pixels with specific brightness levels in different directions (horizontal, vertical, 45°, and 135°) and at different distances of pixel pairs (d = 1, …, 9) [54]. The feature name for gray-level co-occurrence matrix consists of GLCM (features are derived from the symmetric matrix) or GLCH (features are derived from the asymmetric matrix). The following twelve basic features are calculated from each symmetric and asymmetric matrix: area (Area), angular second moment/energy (AngScMom), contrast (Contrast), correlation (Correlat), sum of squares (SumOfSqs), inverse different moment/homogeneity (InvDefMom), summation mean (SumAverg), summation entropy (SumEntrp), summation variance (SumVarnc), entropy (Entropy), differential variance (DifVarnc), and differential entropy (DifEntrp).

### 2.6. Statistical Analysis

Statistical analysis was performed using GraphPad Prism6 software (GraphPad Software Inc., San Diego, CA, USA). The 144 IRT images obtained from twelve horses (*n* = 12) during the six repetitions of exercise (REs; *n* = 6) and two sampling times (BS 0, BS 1; *n* = 2) were analyzed. Data of blood biomarker levels (12 parameters), temperatures (2 features), and image texture features (88 features) were presented in the form of data series, where each horse represented one realization. Values in Supplementary Tables are presented as mean ± standard deviation (SD). Data series were tested independently for univariate distributions using a Shapiro-Wilk normality test. The comparisons between BSs were assessed using a paired *t*-test for Gaussian data and the Wilcoxon matched-pairs signed rank test for non-Gaussian data. The comparisons between REs were assessed using a repeated-measures one-way ANOVA with Geisser-Greenhouse correction, followed by Tukey’s multiple-comparisons test for Gaussian data and the Friedman test, followed by Dunn’s multiple-comparisons test for non-Gaussian data. The significance level was established as *p* < 0.05.

Linear regressions were calculated for the selected blood biomarkers (3 parameters) and 90 IRT image features (2 features of temperature and 88 features of image texture) independently for each ROI. There were four regression equations for a given data series (CPK, AST, WBC, each IRT image feature) and three measures of differences of linearity for given data pairs (CPK and each IRT image feature; AST and each IRT image feature; WBC and each IRT image feature) presented on each plot. Only plots with evidence of linearity were included in the results. All the slopes were significantly non-zero (*p* < 0.0001). Slopes within data pairs were also compared. If the difference between slopes was not significant (*p* > 0.05), a single slope measurement was calculated for all the data, and then the intercepts within data pairs were compared. When differences between the intercepts were not significant (*p* > 0.05), one intercept was calculated for all the data. The features found to have parallel slopes to selected blood markers (WBC, CPK, ALT) were summarized and marked by colors (red, blue, gray) and by letters (R, r, B, Q, H).

## 3. Results

The initial experiment showed that among 2112 returned combinations of color components and features of image texture in the first region of interest (ROI 1), 672 features (31.82%, *p* < 0.05) differed between thermal images obtained before and after a single bout of exercise. To explore further, repeated exercise was evaluated to determine which features of image texture are related to blood biomarkers of exercise effect.

### 3.1. Selection of Blood Biomarkers 

The mean ± SD values of hematological and biochemical blood biomarkers are summarized in Supplementary Table S1 (available online). RBC, HGB, HCT, and LAC were significantly higher (*p* < 0.05) after exercise (BS 1) than before exercise (BS 0) for each of the six repetitions of exercise (REs). However, no differences were found for these biomarkers between REs. Significant differences between REs after exercise were noted for WBC, CPK, and AST. WBC in BS 1 was significantly higher (*p* = 0.006) in the sixth RE (10.2 ± 1.76 × 10^9^/L) as compared to the first RE (8.0 ± 1.28 × 10^9^/L). WBC was also significantly higher after exercise when compared to before exercise in the fourth (BS 1: 9.9 ± 1.70 × 10^9^/L, BS 0: 8.5 ± 1.21 × 10^9^/L, *p* = 0.032), fifth (BS 1: 9.9 ± 1.76 × 10^9^/L, BS 0: 8.5 ± 1.21 × 10^9^/L, *p* = 0.011), and sixth (BS 1: 10.2 ± 1.76 × 10^9^/L, BS 0: 7.8 ± 1.29 × 10^9^/L, *p* = 0.001) REs, respectively. Similarly, CPK in BS 1 was significantly higher (*p* = 0.048) in the sixth RE (294 ± 70.9 U/L) as compared to the first RE (202 ± 85.3 U/L). CPK was significantly higher after exercise than before exercise in the second (BS 1: 209 ± 65.5 U/L, BS 0: 148 ± 40.2 U/L, *p* = 0.008), third (BS 1: 211 ± 50.8 U/L, BS 0: 154 ± 54.5 U/L, *p* = 0.022), fourth (BS 1: 239 ± 102.0 U/L, BS 0: 176 ± 30.8.5 U/L, *p* = 0.025), fifth (BS 1: 245 ± 65.2 U/L, BS 0: 134 ± 30.9 U/L, *p* = 0.0001), and sixth (BS 1: 294 ± 70.9 U/L, BS 0: 148 ± 45.3 U/L, *p* < 0.0001) REs. Moreover, AST was significantly higher (*p* = 0.022) in the sixth RE (309 ± 51.8 U/L) as compared to the first RE (238 ± 47.3 U/L). However, AST was only significantly higher after exercise in the fifth (BS 1: 282 ± 33.8 U/L, BS 0: 263 ± 19.1 U/L, *p* = 0.049) and sixth (BS 1: 309 ± 51.8 U/L, BS 0: 244 ± 43.9 U/L, *p* = 0.018) REs. Therefore WBC, CPK, and AST were selected as the representative blood biomarkers of exercise effect in the investigated model. These biomarkers were further compared with analyzed features of image texture, as well as IRT measures.

### 3.2. Relation of Image Texture with Conventional Blood Biomarkers

Twelve features of image texture in thermal images of the thoracolumbar region were found to have parallel slopes to blood biomarkers in a linear regression model. These features included nine features in the RGB color model, one feature in the YIQ color model (Q.HS.Variance), and two features in the HSB color model (H.HS.Perc99, B.HS.Perc99). Within the RGB color model, eight features were associated with the red component (R.HS.Variance, r.HS.Variance, R.GLCM.SumOfSqs, r.GLCM.SumOfSqs, r.GLCH.SumOfSqs, R.GLCM.SumVarnc, r.GLCM.SumVarnc, r.GLCH.SumVarnc) and one feature with the blue component (B.GLCM.SumVarnc). These features were then compared against IRT measures.

#### 3.2.1. Selection of Features in RGB Color Model

**Figure 3:**
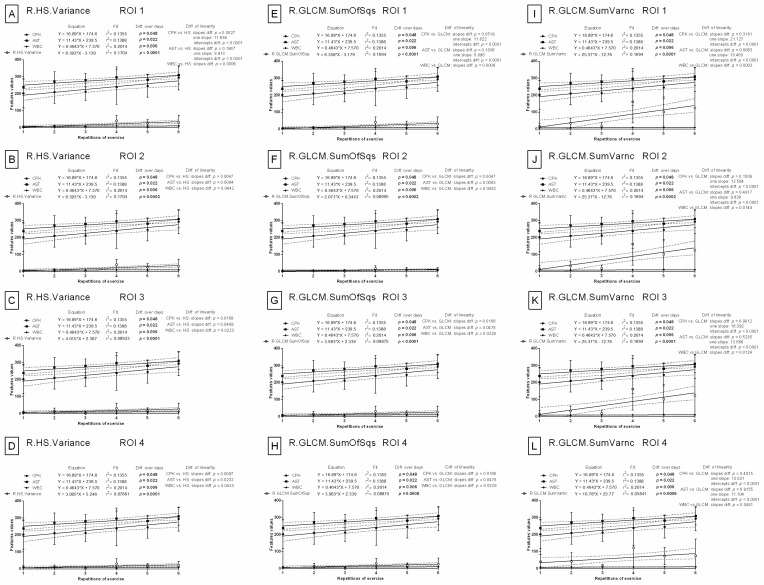
Comparison of image texture features with conventional blood biomarkers in repeated exercises. Features derived from the un-normalized red component in the RGB color model were visualized for ROI 1 (**A**,**E**,**I**), ROI 2 (**B**,**F**,**J**), ROI 3 (**C**,**G**,**K**), and ROI 4 (**D**,**H**,**L**). ROI—region of interest; CPK—creatine phosphokinase activity; AST—aspartate aminotransferase activity; WBC—white blood cell count; R.HS.Variance—variance of histogram statistics, red component in RGB color model (**A**–**D**); R.GLCM.SumOfSqs—sum of squares of gray-level co-occurrence matrix, red component in RGB color model (**E**–**H**); R.GLCM.SumVarnc—sum of variance of gray-level co-occurrence matrix, red component in RGB color model (**I**–**L**). Similarity was tested using linear regressions, and a *p*-value of less than 0.05 was considered significant. If the difference between slopes was not significant, a single slope measurement was calculated for all the data.

The slopes of the linear regression equations for CPK (*p* = 0.0527) and AST (*p* = 0.1867), compared to the slope of the red component variance in the RGB color model (R.HS.Variance), were not significantly different in the first region of interest (ROI 1). However, these differences were significant in all other ROIs. One slop values for CPK vs. R.HS.Variance and AST vs. R.HS.Variance were 11.639 and 8.913, respectively (Figure 3A–D). With respect to the slope of the sum-of-squares values for the gray-level co-occurrence matrix of the red component in the RGB color model (R.GLCM.SumOfSqs), the slopes of CPK (*p* = 0.0519) and AST (*p* = 0.1836) were not significantly different in ROI 1. However, these differences were significant in all other ROIs. One slop values were 11.622 (CPK vs. R.GLCM.SumOfSqs) and 8.896 (CPK vs. R.GLCM.SumOfSqs) (Figure 3E–H). With respect to the sum of variance values of the gray-level co-occurrence matrix of the red component in the RGB color model (R.GLCM.SumVarnc), no significant differences in the slopes for CPK and AST were noted in ROI 1 (CPK *p* = 0.3161, one slope: 21.127; AST *p* = 0.0663, one slope: 18.400), ROI 2 (CPK *p* = 0.1509, one slope: 12.564; AST *p* = 0.4917, one slope: 9.838), ROI 3 (CPK *p* = 0.9012, one slope: 16.392; AST *p* = 0.5235, one slope: 13.666), and ROI 4 (CPK *p* = 0.4015, one slope: 13.831; AST *p* = 0.9155, one slope: 11.104) (Figure 3I–L). 

**Figure 4:**
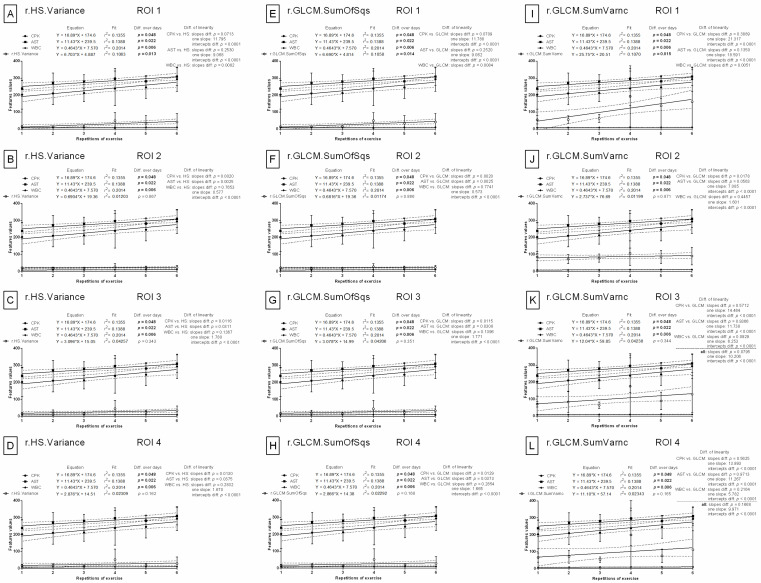
Comparison of image texture features with conventional blood biomarkers in repeated exercises. Features derived from the normalized red component in the RGB color model were visualized for ROI 1 (**A**,**E**,**I**), ROI 2 (**B**,**F**,**J**), ROI 3 (**C**,**G**,**K**), and ROI 4 (**D**,**H**,**L**). ROI—region of interest; CPK—creatine phosphokinase activity; AST—aspartate aminotransferase activity; WBC—white blood cell count; r.HS.Variance—normalized variance of histogram statistics, red component in RGB color model (**A**–**D**); r.GLCM.SumOfSqs—normalized sum of squares of gray-level co-occurrence matrix, red component in RGB color model (**E**–**H**); r.GLCM.SumVarnc—normalized sum of variance of gray-level co-occurrence matrix, red component in RGB color model (**I**–**L**). Similarity was tested using linear regressions. A *p*-value of less than 0.05 was considered significant. If the difference between slopes was not significant, a single slope measurement was calculated for all the data.

The slopes of the linear regression were not significantly different for either CPK or AST when compared to the slopes of the normalized variance (r.HS.Variance) or the normalized sum of squares of the gray-level co-occurrence matrix (r.GLCM.SumOfSqs) of the red component in the RGB color model in ROI 1 (CPK vs. r.HS.Variance *p* = 0.0713, one slope: 11.795; AST vs. r.HS.Variance *p* = 0.2530, one slope: 9.068; (Figure 4A)) (CPK vs. r.GLCM.SumOfSqs *p* = 0.0709, one slope: 11.788; AST vs. r.GLCM.SumOfSqs *p* = 0.2520, one slope: 9.062; (Figure 4E)). In all other ROIs, the slopes were not significantly different (*p* > 0.05) for WBC vs. r.HS.Variance (Figure 4B–D), as well as WBC vs. r.GLCM.SumOfSqs (Figure 4F–H). Moreover, the normalized sum of variance of gray-level co-occurrence matrix of the red component in the RGB color model (r.GLCM.SumVarnc) did not yield any significant differences for any blood biomarkers in ROI 3 (*p* = 0.0795, one slope: 10.206; (Figure 4K)) or ROI 4 (*p* = 0.1668 one slope: 9.971; (Figure 4L)). In ROI 1, the slopes were not significantly different for either CPK (*p* = 0.3889, one slope: 21.317) or AST (*p* = 0.1350, one slope: 18.591) when compared to the slope of r.GLCM.SumVarnc (Figure 4I). In ROI 2, neither the slope of AST (*p* = 0.0563, one slope: 7.085) nor WBC (*p* = 0.4457, one slope: 1.601) was significantly different when compared to r.GLCM.SumVarnc (Figure 4J).

**Figure 5:**
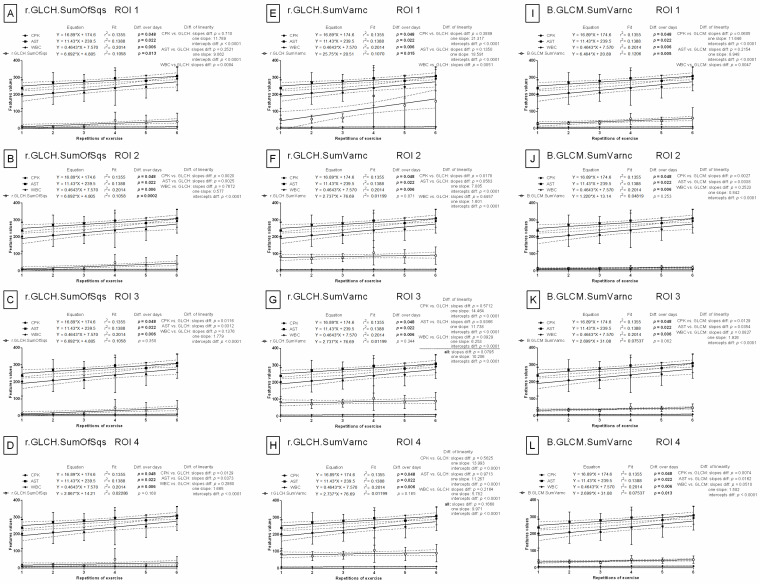
Comparison of image texture features with conventional blood biomarkers in repeated exercises. Features derived from the normalized red component and the un-normalized blue component in the RGB color model were visualized for ROI 1 (**A**,**E**,**I**), ROI 2 (**B**,**F**,**J**), ROI 3 (**C**,**G**,**K**), and ROI 4 (**D**,**H**,**L**). ROI—region of interest; CPK—creatine phosphokinase activity; AST—aspartate aminotransferase activity; WBC—white blood cell count; r.GLCH.SumOfSqs—normalized sum of squares of the asymmetric gray-level co-occurrence matrix, red component in RGB color model (**A**–**D**); r.GLCH.SumVarnc—normalized sum of variance of the asymmetric gray-level co-occurrence matrix, red component in RGB color model (**E**–**H**); B.GLCM.SumVarnc—sum of variance of gray-level co-occurrence matrix, blue component in RGB color model (**I**–**L**). Similarity was tested using linear regressions. A *p*-value of less than 0.05 was considered significant. If the difference between slopes was not significant, a single slope measurement was calculated for all the data.

The slopes of linear regression were not significantly different for either CPK (*p* = 0.710, one slope: 11.789) or AST (*p* = 0.2521, one slope: 9.062) when compared to the slope of the normalized sum of squares of the asymmetric gray-level co-occurrence matrix of the red component in the RGB color model (r.GLCH.SumOfSqs) in ROI 1 (Figure 5A). The only significant differences were found with WBC (*p* > 0.05) in other ROIs (Figure 5B–D). Similarly, the slopes were not significantly different for either CPK (*p* = 0.3889, one slope: 21.317) or AST (*p* = 0.1350, one slope: 18.591) as compared to the normalized sum of variance of the asymmetric gray-level co-occurrence matrix of the red component in the RGB color model (r.GLCH.SumVarnc) in ROI 1 (Figure 5E). The slopes in ROI 3 (*p* = 0.0795, one slope: 10.206; (Figure 5G)) and ROI 4 (*p* = 0.1668 one slope: 9.971; (Figure 5H)) were not significantly different for any selected blood biomarkers. In ROI 2, the slopes were not significantly different for either AST (*p* = 0.0563, one slope: 7.083) or WBC (*p* = 0.4457, one slope: 1.601) when compared to the slope of r.GLCH.SumVarnc (Figure 5F). In contrast to the eight features of the red component in the RGB color model, only the slope of one feature of the blue component, the sum of variance of gray-level co-occurrence matrix (B.GLCM.SumVarnc), was found not to be significantly different from that of the blood biomarkers in all ROIs. In ROI 1, slopes were parallel for linear regressions of CPK (*p* = 0.0605, one slope: 11.646) and AST (*p* = 0.2154, one slope: 8.948) (Figure 5I), whereas in other ROIs, only WBC (*p* > 0.05) (Figure 5J–L) was found to have a parallel slope. All the remaining slopes from the 528 feature/CPK pairs, 528 feature/AST pairs, and 528 feature/WBC pairs in the RGB color model were significantly different (*p* < 0.05).

#### 3.2.2. Selection of Features in YUV and YIQ Color Models

**Figure 6:**
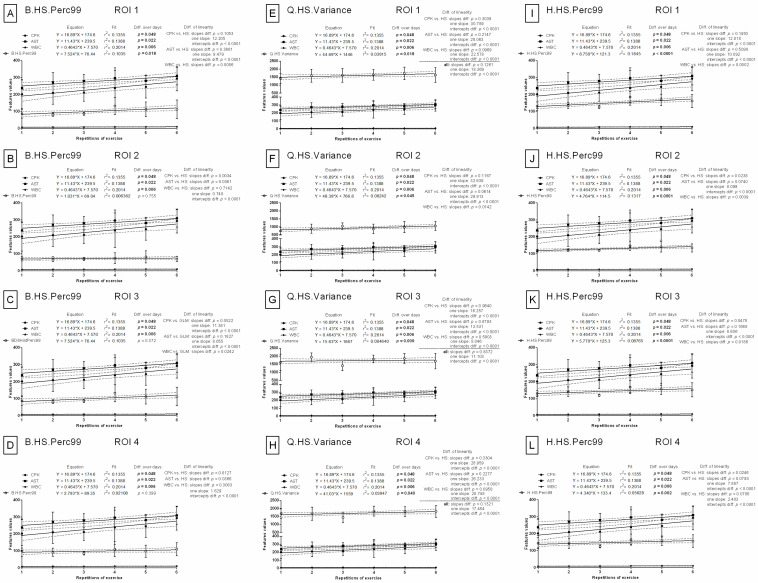
Comparison of image texture features with conventional blood biomarkers in repeated exercises. Features derived from the un-normalized Q component in the YIQ color model, as well as the un-normalized Hue component and un-normalized Brightness component in the HSB color model, were visualized for ROI 1 (**A**,**E**,**I**), ROI 2 (**B**,**F**,**J**), ROI 3 (**C**,**G**,**K**), and ROI 4 (**D**,**H**,**L**). ROI—region of interest; CPK—creatine phosphokinase activity; AST—aspartate aminotransferase activity; WBC—white blood cell count; Q.HS.Variance—variance of histogram statistics, Q component in YIQ color model (**A**–**D**); H.HS.Perc99—99th percentiles of histogram statistics, hue component in HSB color model (**E**–**H**); B.HS.Perc99—99th percentiles of histogram statistics, brightness component in HSB color model (**I**–**L**). Similarity was tested using linear regressions. A *p*-value of less than 0.05 was considered significant. If the difference between slopes was not significant, a single slope measurement was calculated for all the data.

Only for one feature of the YIQ color model, variance of the Q component (Q.HS.Variance), were the slopes found not to be significantly different when compared to blood biomarkers in three out of four ROIs. In ROI 1 (*p* = 0.1261, one slope: 18.369), ROI 3 (*p* = 0.8372, one slope: 11.103), and ROI 4 (*p* = 0.1321, one slope: 17.454), the combined slope for all data was not significantly different. In ROI 2, only the slopes of CPK (*p* = 0.1137, one slope: 32.639) and AST (*p* = 0.0614, one slope: 29.913) were found not to be significant (Figure 6A–D). All the remaining slopes from the 528 feature/CPK pairs, 528 feature/AST pairs, and 528 feature/WBC pairs in each of the YUV and YIQ color models were significantly different (*p* < 0.05).

#### 3.2.3. Selection of Features in HSB Color Model

The slopes of the linear regression equations were not significantly different for two features in the HSB color model when compared to a single blood biomarker in selected ROIs. In ROI 1, no significant differences were noted for either CPK or AST when compared to the 99th percentiles of either the hue component (H.HS.Perc99) or the brightness component (B.HS.Perc99) (CPK vs. H.HS.Perc99 *p* = 0.1450, one slope: 12.818; AST vs. H.HS.Perc99 *p* = 0.5090, one slope: 10.092; (Figure 6E)) (CPK vs. B.HS.Perc99 *p* = 0.1053, one slope: 12.205; AST and B.HS.Perc99 *p* = 0.3651, one slope: 9.479; (Figure 6I)). In ROI 2, no differences were found between the slopes of AST vs. H.HS.Perc99 (*p* = 0.0740, one slope: 8.099; (Figure 6F)) or WBC vs. B.HS.Perc99 (*p* = 0.7142, one slope: 0.748; (Figure 6J)). In ROI 3, no significant differences were noted when comparing the slope of AST with either HS.Perc99s (AST vs. H.HS.Perc99 *p* = 0.1668, one slope: 8.606 (Figure 6G)) or B.HS.Perc99 (*p* = 0.1827, one slope: 8.655). The same is true when comparing CPK with B.HS.Perc99 (*p* = 0.0522, one slope: 11.381 (Figure 6K)). In ROI 4, no significant differences were observed between the slope comparisons of WBC with HS.Perc99s (*p* > 0.05, one slope: 8.606 (Figure 6H,L)) or AST with H.HS.Perc99 (*p* = 0.0793, one slope: 7.887 (Figure 6H)). All the remaining slopes from the 528 features/CPK pairs, 528 features/AST pairs, and 528 feature/WBC pairs in the HSB color model were significantly different (*p* < 0.05).

### 3.3. Comparison of Image Texture with IRT Measures

**Figure 7:**
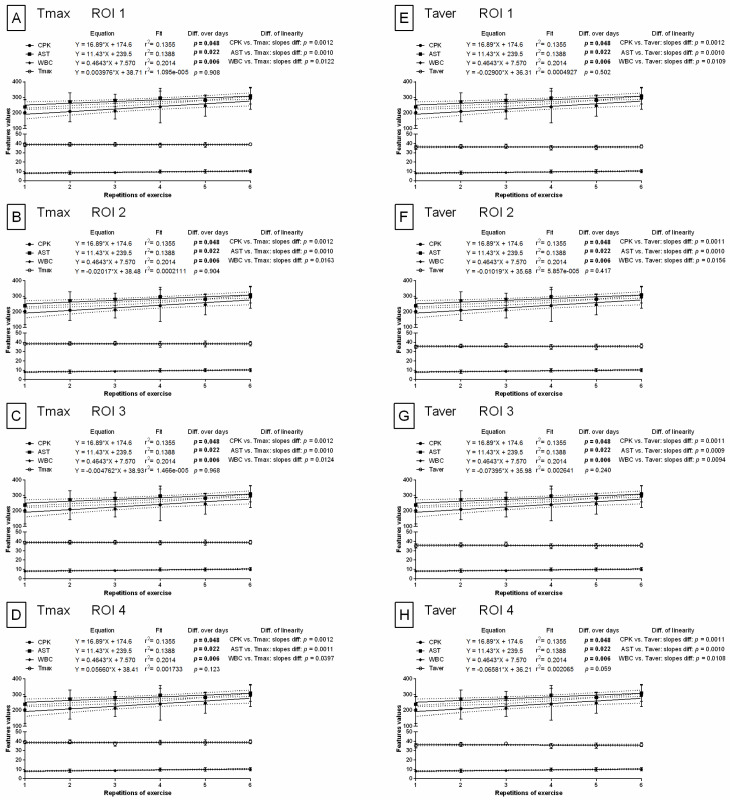
Comparison of body surface temperatures with conventional blood biomarkers in repeated exercises. Conventional IRT measures were visualized for ROI 1 (**A**,**E**), ROI 2 (**B**,**F**), ROI 3 (**C**,**G**), and ROI 4 (**D**,**H**). ROI—region of interest; CPK—creatine phosphokinase activity; AST—aspartate aminotransferase activity; WBC—white blood cell count; Tmax—maximal temperature (**A**–**D**); Taver—average temperature (**E**–**H**). Similarity was tested using linear regressions. A *p*-value of less than 0.05 was considered significant.

In contrast to the features of image texture analysis, the slopes in the linear regression models were significantly different (*p* < 0.05) for all blood biomarkers when compared to either conventional IRT measures (maximal temperature (Tmax) (Figure 7A–D) or average temperature (Taver) (Figure 7E–H)). No signs of relationships between blood biomarkers and conventional IRT measures over repetitions of exercise were observed in any examined ROIs.

The mean ± SD values of conventional IRT measures are summarized in Supplementary Table S2 (available online). Tmax and Taver were significantly higher (*p* < 0.0001) after exercise than before exercise at each of the six repetitions of exercise and in all examined ROIs. However, no differences were found for either Tmax or Taver between REs. For comparison, the mean ± SD values for selected features of image texture are summarized in Supplementary Table S3 (available online). In ROI 1, R.HS.Variance, r.HS.Variance, R.GLCM.SumOfSqs, r.GLCM.SumOfSqs, and r.GLCH.SumOfSqs increased significantly (*p* < 0.0001) with REs after effort and differed significantly (*p* < 0.05) between BS 0 and BS 1 in all REs. R.GLCM.SumVarnc, r.GLCM.SumVarnc, and r.GLCH.SumVarnc differed significantly (*p* < 0.05) between BS 0 and BS 1 in ROI 1, ROI 3, and ROI 4 but not in ROI 2. B.GLCM.SumVarnc in ROI 1 increased significantly (*p* = 0.0005) with REs after effort; however, BS 0 and BS 1 only differed significantly (*p* < 0.05) in the first, fourth, and fifth REs. Q.HS.Variance was highly related to the examined effort model in all four examined ROIs. Both significantly increased (*p* < 0.05) with REs after effort, and significant differences (*p* < 0.05) between BSs in all REs were observed without exception. H.HS.Perc99 increased significantly (*p* < 0.05) with repeated REs and differed significantly (*p* < 0.05) between BS 0 and BS 1 in most REs in ROI 1, ROI 3, and ROI 4 but not in ROI 2. B.HS.Perc99 differed significantly (*p* < 0.05) between BS 0 and BS 1 in most REs in ROI 1, ROI 3, and ROI 4.

**Figure 8:**
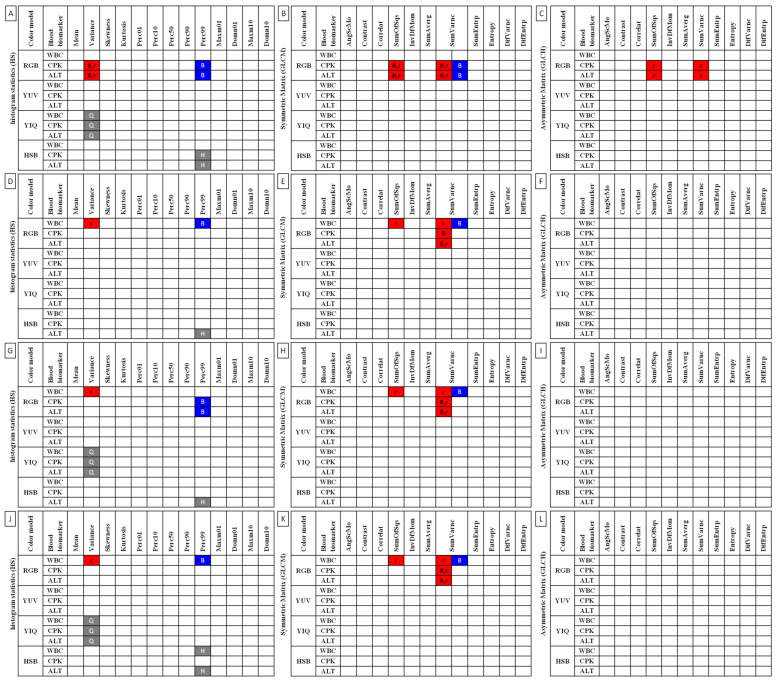
Features of histogram statistics (**A**,**D**,**G**,**J**), symmetric gray-level co-occurrence matrix (**B**,**E**,**H**,**K**), and asymmetric gray-level co-occurrence matrix (**C**,**F**,**I**,**L**) for examined color models (RGB, YUV, YIQ, HSB) found to have parallel slopes to selected blood markers (WBC, CPK, ALT) in (**A**–**C**) ROI 1, (**D**–**F**) ROI 2, (**G**–**I**) ROI 3, and (**J**–**L**) ROI 4. Parallel slopes are marked with different colors and letters for different color components. R, r—red component in the RGB color model; B—blue component in the RGB color model; Q—Q component in the YIQ color model; H—hue component in the HSB color model. Capital letters (R, B, Q, H) annotate the un-normalized channels, whereas a lowercase letter (r) annotates the normalized channel.

In summary, twelve features of image texture had parallel slopes to blood markers in ROI 1, and three variants of SumVarnc (R.GLCM.SumVarnc, r.GLCM.SumVarnc, r.GLCH.SumVarnc) were found to have parallel slopes to blood markers in ROIs 3 and 4. Q.HS.Variance was the only feature with parallel slopes to blood markers in all ROIs; all should be considered for further research (Figure 8).

## 4. Discussion

Data of the current and related studies show that the IRT has the potential to allow for the monitoring of workload in horses [15,19,26,27,28,39,55,56]. However, many factors affecting the results, reliability, and repeatability of the IRT examination make this technique very speculative [24,25,57]. Therefore, the current study focused on the introduction of advanced image texture analysis for the description of exercise effort [58]. Further work on this topic should examine whether IRT allows for monitoring of increasing levels of workload. Knowing the limitations that can be encountered during conventional IRT, further image analyses are necessary [19,24,25,26,27,28,57]. Conversion of color images into the three basic RGB components and the more advanced analytic approach allow for better use of IRT images. Advanced IRT analyses with the simultaneous evaluation of blood biomarkers in the multiple-exercises model would allow for differentiation of the effort and load of a horse during exercise [4,6,7,15,27,39,56,57,58]. The use of increasing exercise intensity rather than the same exercise test within six following days would be an unjustified omission of the necessary stage of implementation work. This would lead to an excessive number of variables that would be difficult to interpret properly. Some blood variables and texture IRT features change over time, especially after exercise tests. This phenomenon could be useful in detecting the need to continue exercise up to a certain level of effort during training to provide an expected conditioning response [2,5,12]. Among the examined blood biomarkers, WBC, CPK, and AST increased over multiple exercise trials. The increase in WBC may be caused by higher cortisol concentration, as the redistribution of WBC from the marginal pool is mostly regulated by cortisol release [59,60]. The exercise-intensity-dependent cortisol increase usually reaches a peak 15–30 min after the beginning of the exercise [59,60] and regulates WCB migration, crucial in homeostatic conditions [61,62]. Widely used blood biomarkers of muscle damage, CPK and AST are cumulative; thus, their steady increase across six days of multiple exercise trials seems informative [63,64]. After effort, a horse’s body needs at least 24 h to clear the bloodstream of CPK and AST [4,16], excreting CPK faster than AST [4]. CPK and AST levels after effort depend on the effort intensity [59,60,61,62,63,64,65], conditioning and type of usage [61,62,65], as well muscle mass and composition [61,62]. This feature indicates their possible use in further research on increased exercise intensity. The parallelism of WBC, CPK, and ALT slopes and variance; 99th percentiles; sum of squares; and summation variance with consecutive components in RGB, YIQ, and HSB color models was shown in the research. This shows the possibility of predicting an increase in blood biomarkers based on the texture features of noninvasive IRT imaging. Basic thermal camera software for smartphones and more advanced software for medical applications are developing rapidly [66,67,68,69]. We believe our advances in the IRT equine load-monitoring approach could be easily and accurately transferred into typical daily riding practice as a tool for horse owners, competitors, and trainers. Such an approach can be used to monitor the exertion level of a horse to achieve the required training response without tipping over into fatigue levels associated with injury. However, the introduction of advanced digital IRT image processing into the monitoring of equine welfare during conditioning and competition requires further research, including the assessment of equine emotional state [70,71,72,73,74,75,76] and cortisol release [72,75,76].

A color model is a model that uses mathematical functions to convert light-color coordinates into three color components in three-dimensional space [77,78]. In the current research, RGB color cube, YUV and YIQ color spaces, and HSB color coin (Figure 1) were the models considered. As the color model is the digital representation of possibly contained colors [77], a different color model may convey differing features of image texture. In the current study, the most potentially informative features were observed in the RGB color model. This finding is consistent with the basic IRT rainbow palette, where high temperature is red-annotated and low temperature is blue-annotated [19,79]. After physical effort, whether single or repeated, body surface temperature increases [15,26,27,39], and as a result, the count of red-annotated pixels in IRT images also increases. Moreover, in the RGB color model, the acquired image does not need any further transformation to display features of image texture. Therefore, the RGB color model is considered the default color model for most image applications [27,28,29,30,31,32,33,34,36,39,42], and researchers do not typically investigate other available color models. Our findings support the selection of the RGB color model as a default color model for the analysis of image texture of equine IRT images. However, feature extraction in the YIQ and potentially the HSB color models should also be considered. The variance of the Q component in the YIQ color model was the only feature related to all blood biomarkers in three out of four ROIs. As human vision can recognize two forms of images, RGB images and grayscale images, the YUV and YIQ color models were developed to provide compatibility between these two forms [77]. The YIQ color model differs from YUV in that it emphasizes sensitivity to changes in luminance rather than hue or saturation changes [80].

In the YIQ color model, the Y component represents brightness, the I component corresponds to the orange-cyan axis, and the Q component corresponds to the magenta-green axis [77,80]. This is consistent with the basic IRT rainbow palette, where the medium-high temperature is magenta-annotated and the medium-low temperature is green-annotated [19,79]. When body surface temperature increases after effort [15,26,27,39], the count of magenta-annotated pixels in the IRT image also increases. We suspect that the increase in medium-high temperature on the IRT image may be successfully quantified in the YIQ color model using digital image analysis of IRT images. However, further research is required. Therefore, not including the transformation of IRT images to the YIQ color model in further equine applications may result in the loss or inadvertent omission of important image texture data. Our findings suggest that the YIQ color model is an additional informative color model that should be considered in subsequent digital IRT image processing in equine applications. The HSB color model is based on the human visual system [80]. In this investigation, it exhibited a poor repeatability between ROIs, correlation with few blood biomarkers, and differentiation between BSs and REs. In the HSB color model, the brightness component is separated from the hue component and saturation component [78], whereas in the YIQ color model, the hue and saturation are the latter components [80]. It seems that representation of the same signal for both color and grayscale images using the brightness component in YIQ color model [77] is more suitable to digital IRT image processing than the separation of the brightness component in the HSB color model [78]. The selection of the YIQ and HSB color models, as additive to the RGB model, can be considered useful in effort-dependent assessment, similar to pregnancy detection [58]. Therefore, the RGB, YIQ, and HSB color models should be carefully considered for subsequent digital IRT image processing in equine applications.

Only two of the applied computational gray-level matrices (both symmetric GLCM and asymmetric GLCH variants) provided features of an image texture with slopes parallel with conventional reference measures of exercise effort. Our findings stand partially in agreement with and partially in contradiction to recent research conducted on the single-exercise effort model [39,56]. In recent research, not only GLCM but also GRLM features demonstrated effort-dependent differences [39,56]. In this study, no evidence of similarity was noted for blood biomarkers and GRLM features. Interestingly, differing features of GLCM were informative in the single-effort [39,56] compared to the repeated-effort research model. In the single-effort model, features of contrast (Contras, Correlat [39]) and order (Entropy, DifEntrp, DifVarnc, InvDefMom [39,56], SumEntrp [56]) were found to be informative, whereas in the current study, features of variance (SumOfSqs, SumVarnc) were informative. Moreover, features of variance (Variance) in histogram statistics were in line with blood biomarker measures. Therefore, it can be carefully assumed that the variability of IRT image texture may be a promising and novel tool for the detection of exercise effort. However, extensive investigations are required to fully understand its potential applications.

All eight features of the variability of the red component in the RGB color model were highly repeatable between image-processing protocols. The results of Variance, SumOfSqs, and SumVarnc were repeatable, whether the images were normalized (r) or not (R). Likewise, the results of normalized SumOfSqs and SumVarnc were repeatable whether the symmetric (GLCM) or asymmetric (GLCH) matrices were applied. Additionally, un-normalized SumVarnc of GLCM was related to blood biomarkers in the examined effort markers, as was also observed in the blue component of the RGB color model. These highly repeatable findings justify the selection of the RGB color model as the default color model for further equine IRT applications, which is in agreement with recently reported results [56,57]. In addition, histogram statistics and gray-level co-occurrence matrix are suggested approaches for analysis of image texture data.

The thoracolumbar region was selected as the examined thermal window based on recent research, as equine neck and trunk regions were recognized as the most suitable for the determination of thermal effects of under-saddle work [15,81]. IRT limitations are related to the effect of ambient temperature [24,25,82,83], the warming effect of sunlight exposure [19,57,82], and the cooling effect of airflow during movement [27,84]. The under-saddle region was chosen as the least susceptible to the influence of external conditions in the current and recent research [39,56]. Texture features of IRT images from ROI 1, representing the withers area of the thoracolumbar region, provided the most consistent correlation with blood biomarkers. ROI 1 is a small area symmetrically covering the left and right cranial part of the back muscles and the first vertebrae of the thoracic spine [85]. ROI 1 is suitable for GLCM and GLRLM best image-segmentation conditions. Those conditions encompass annotation of a small and functionally differential area to detect small lesions in low-resolution medical images [35]. Likewise, three variants of SumVarnc were related to blood biomarker levels in ROI 3 and ROI 4, representing a large area of back muscles on the left and right. The previously discussed repeatability of SumVarnc and the consistent measurements obtained in the large areas covering the back muscles indicates the potential applicability of this feature for the evaluation of IRT images. Since approximately 70%–80% of the energy produced during exercise by working muscles is released as heat [84], the size of the muscular unit underlying the imaged area significantly affects the measurement of body surface temperature [15,19,26,27]. Recent research has found that the highest temperature values after single-effort exercise were observed in the areas covering three large thoracolumbar muscles (*m. latissimus dorsi*, *m. obliquus externus abdominis*, and *m. pectoralis transversus*) [15]. However, in this study the complete segmentation of the horse’s body surface in terms of muscle units was not taken into account, similarly to other studies. More complete segmentation was proposed in the interspecific comparative studies of equids [38]; however, the effect of exercise was not considered. The presence of structures not producing heat, such as bones lying directly under the imaged area in IRT, oppositely affects the measurement results [55,58,86]. Therefore, it is not surprising that no significant results were observed in ROI 2, which represents the thoracic spine area with the least influence of back muscles [58]. Only Q.HS.Variance was related to blood markers in the examined effort model in four ROIs, regardless of the mass of muscles lying under the examined areas.

## 5. Conclusions

Based on the current preliminary results, it can be concluded that the combination of two analytical approaches to image texture (histogram statistics and gray-level co-occurrence matrix) and two color models of thermal images (RGB and YIQ) should be strongly considered as the most appropriate digital image-processing methods applicable in equine IRT. Furthermore, the experimental results suggest that the features of image texture (variance, sum of square, and sum of variance) found to be informative of the effect of exercise in horses must be investigated extensively in further equine applications. We plan to extend the present preliminary experiments by using other initial experimental designs, i.e., other muscle areas, effort intensity, types of work (jumping, dressage, eventing, endurance, and racing), etc. Current research should be treated as preliminary because further experiments should be conducted involving equine athletes. This would allow for more specific conclusions to be drawn regarding exercise effectiveness and avoidance of excessive fatigue. The long-term objective is to determine the most appropriate combinations of color models and the best approach to digital image processing for the effective detection of exercise effect in equine athletes at peak competition levels.

## Data Availability

The data presented in this study are available on request from the corresponding author.

## Supplementary Materials

The following are available online at https://www.mdpi.com/article/10.3390/ani12040444/s1, Table S1: Blood biomarkers level (mean ± SD) before (BS 0) and after (BS 1) exercise in the model of six repetitions of exercise (REs). Table S2: Body surface temperature (mean ± SD) in four regions of interest (ROIs 1-4) before (BS 0) and after (BS 1) exercise in the model of six repetitions of exercise (REs). Table S3: Selected features of image texture analysis (mean ± SD) in four regions of interest (ROIs 1-4) before (BS 0) and after (BS 1) exercise in the model of six repetitions of exercise (REs).

## Abbreviations

The following abbreviations are used in this manuscript:

ALT	Alanine aminotransferase
AM	Autoregressive model
AngScMom	Angular second moment/energy
AST	Aspartate aminotransferase
B	Blue component in the RGB color model
B	Brightness component in the HSB color model
BS	Blood sampling
Correlat	Correlation
CPK	Creatine phosphokinase
DifEntrp	Differential entropy
DifVarnc	Differential variance
e	Emissivity
EMG	Electromyography
G	Green component in the RGB color model
GLCH	Asymmetric gray-level co-occurrence matrix
GLCM	Symmetric gray-level co-occurrence matrix
GLevNonUn	Gray-level non-uniformity
GLM	Gray-level matrix
GM	Gradient map
GradArea	Absolute gradient area
GradMean	Absolute gradient mean
GradNonZeros	Percentage of pixels with nonzero gradient
GradSkewness	Absolute gradient skewness
GradVariance	Absolute gradient variance
GRLM	Gray-level run-length matrix
GT	Gabor transform
H	Hue component in the HSB color model
HCT	Hematocrit
HGB	Hemoglobin concentration
HistArea	Histogram area
HistDomn	Histogram dominants
HistKurtosis	Histogram kurtosis
HistMaxm	Histogram maximum of moments
HistMean	Histogram mean
HistPerc	Histogram percentile
HistSkewness	Histogram skewness coefficient
HistVariance	Histogram variance
HOG	Histogram of oriented gradients
HR	Heart rate
HRV	Heart rate variability
HS	Histogram statistics
HSB	Hue/Saturation/Brightness color model
I	I component in the YIQ color model
II	Infrared thermography imaging
InvDefMom	Inverse different moment/homogeneity
IRT	Infrared termography
LAC	Lactate concentration
LngREmph	Moment of long string emphasis
MCH	Mean corpuscular hemoglobin
MCHC	Mean corpuscular hemoglobin concentration
MCV	Mean corpuscular volume
MGLevNonUn	Gray-level non-uniformity moment
MRLNonUni	Run-length nonuniformity moment
Q	Q component in the YIQ color model
R	Red component in the RGB color model
RBC	Red blood cells count
RE	Repetition of exercise
RGB	Red/Green/Blue color model
RH	Relative humidity
RLNonUni	Run-length nonuniformity
ROI	Region of interests
S	Saturation component in the HSB color model
SET	Standardized exercise test
ShrtREmp	Reverse moment of short string emphasis
SumAverg	Summation mean
SumEntrp	Summation entropy
SumOfSqs	Sum of squares
SumVarnc	Summation variance
Taver	Average temperature
Tmax	Maximal temperature
TSP	Total serum protein
U	U component in the YUV color model
WBC	White blood cell count
v	Velocity
V	V component in the YUV color model
Y	Brightness in the YUV and YIQ color models
YIQ	Brightness/I component/Q component color model
YUV	Brightness/U component/V component color model

## References

[1] Goachet A.G., Julliand V. (2015). Implementation of field cardio-respiratory measurements to assess energy expenditure in Arabian endurance horses. Animal.

[2] Webb H., Weston J., Norman E., Cogger N., Rogers C. (2019). Experience, riding practices and training methods of fédération equestre internationale (fei: 80–160 km) level endurance horse rider-owner-trainers in new zealand. Comp. Exerc. Physiol..

[3] Murphy B.A. (2019). Circadian and circannual regulation in the horse: Internal timing in an elite athlete. J. Equine Vet. Sci..

[4] Mami S., Khaje G., Shahriari A., Gooraninejad S. (2019). Evaluation of biological indicators of fatigue and muscle damage in arabian horses after race. J. Equine Vet. Sci..

[5] Cottin F., Barrey E., Lopes P., Billat V. (2006). Effect of repeated exercise and recovery on heart rate variability in elite trotting horses during high intensity interval training. Equine Vet. J..

[6] Souza L., Hunka M.M., Nery P.C.R., Coelho C.S., Manso H.E.C., Manso Filho H.C. (2018). The effect of repeated barrel racing on blood biomarkers and physiological parameters in quarter horses. Comp. Exerc. Physiol..

[7] Kinnunen S., Atalay M., Hyyppa S., Lehmuskero A., Hanninen O., Oksala N. (2015). Effects of prolonged exercise on oxidative stress and antioxidant defense in endurance horse. J. Sports Sci. Med..

[8] Gondim F.J., Zoppi C.C., dos Reis Silveira L., Pereira-da Silva L., de Macedo D.V. (2009). Possible relationship between performance and oxidative stress in endurance horses. J. Equine Vet. Sci..

[9] Takahashi Y., Takahashi T., Mukai K., Ohmura H. (2021). Effects of fatigue on stride parameters in thoroughbred racehorses during races. J. Equine Vet. Sci..

[10] Takahashi Y., Mukai K., Ohmura H., Takahashi T. (2021). Changes in muscle activity with exercise-induced fatigue in thoroughbred horses. Comp. Exerc. Physiol..

[11] Hodgson D.R., McGowan C.M., McKeever K. (2013). The Athletic Horse: Principles and Practice of Equine Sports Medicine.

[12] Arfuso F., Gianetto C., Giudice E., Fazio F., Panzera M., Piccione G. (2021). Peripheral modulators of the central fatigue development and their relationship with athletic performance in jumper horses. Animals.

[13] Lewis V., Kennerley R. (2017). A preliminary study to investigate the prevalence of pain in elite dressage riders during competition in the united kingdom. Comp. Exerc. Physiol..

[14] Kirsch K., Düe M., Holzhausen H., Sandersen C. (2019). Correlation of competition performance with heart rate and blood lactate response during interval training sessions in eventing horses. Comp. Exerc. Physiol..

[15] Witkowska-Piłaszewicz O., Maśko M., Domino M., Winnicka A. (2020). Infrared thermography correlates with lactate concentration in blood during race training in horses. Animals.

[16] Wan J.-J., Qin Z., Wang P.-Y., Sun Y., Liu X. (2017). Muscle fatigue: General understanding and treatment. Exp. Mol. Med..

[17] Lenoir A., Trachsel D.S., Younes M., Barrey E., Robert C. (2017). Agreement between electrocardiogram and heart rate meter is low for the measurement of heart rate variability during exercise in young endurance horses. Front. Vet. Sci..

[18] Williams J.M. (2018). Electromyography in the horse: A useful technology?. J. Equine Vet. Sci..

[19] Soroko M., Howell K. (2018). Infrared thermography: Current applications in equine medicine. J. Equine Vet. Sci..

[20] Colborne G., Birtles D., Cacchione I. (2001). Electromyographic and kinematic indicators of fatigue in horses: A pilot study. Equine Vet. J..

[21] Mohr E., Witte E., Voss B. (2000). Heart rate variability as stress indicator. Arch. Tierz..

[22] Mott R.O., Hawthorne S.J., McBride S.D. (2020). Blink rate as a measure of stress and attention in the domestic horse (equus caballus). Sci. Rep..

[23] Takahashi T., Ohmura H., Mukai K., Matsui A., Aida H. (2014). Fatigue in the superficial and deep digital flexor muscles during exercise in t horoughbred horses. Equine Vet. J..

[24] Satchell G., McGrath M., Dixon J., Pfau T., Weller R. (2015). Effects of time of day, ambient temperature and relative humidity on the repeatability of infrared thermographic imaging in horses. Equine Vet. J..

[25] Maśko M., Witkowska-Piłaszewicz O., Jasiński T., Domino M. (2021). Thermal features, ambient temperature, and hair coat lengths: Limitations of infrared imaging in pregnant primitive breed mares over the year. Reprod. Domest. Anim..

[26] Quesada J.I.P. (2017). Application of Infrared Thermography in Sports Science.

[27] Soroko M., Śpitalniak-Bajerska K., Zaborski D., Poźniak B., Dudek K., Janczarek I. (2019). Exercise-induced changes in skin temperature and blood parameters in horses. Arch. Anim. Breed..

[28] Eddy A., Van Hoogmoed L., Snyder J. (2001). The role of thermography in the management of equine lameness. Vet. J..

[29] Chrysafi A., Athanasopoulos N., Siakavellas N. (2017). Damage detection on composite materials with active thermography and digital image processing. Int. J. Therm. Sci..

[30] Deane S., Avdelidis N.P., Ibarra-Castanedo C., Zhang H., Nezhad H.Y., Williamson A.A., Mackley T., Davis M.J., Maldagu X., Tsourdos A. (2019). Application of NDT thermographic imaging of aerospace structures. Infrared Phys. Technol..

[31] Tejedor B., Barreira E., Almeida R.M., Casals M. (2021). Automated data-processing technique: 2d map for identifying the distribution of the u-value in building elements by quantitative internal thermography. Autom. Constr..

[32] Mancilla R.B., Daul C., Gutierrez Martinez J., Vera Hernandez A., Wolf D., Leija Salas L. Detection of sore-risk regions on the foot sole with digital image processing and passive thermography in diabetic patients. Proceedings of the 17th International Conference on Electrical Engineering, Computing Science and Automatic Control (CCE).

[33] Benjumea E., Morales Y., Torres C., Vilardy J. (2019). Characterization of thermographic images of skin cancer lesions using digital image processing. J. Phys. Conf. Ser..

[34] Silva T.A.E., Silva L.F., Muchaluat-Saade D.C., Conci A. (2020). A computational method to assist the diagnosis of breast disease using dynamic thermography. Sensors.

[35] Depeursinge A., Al-Kadi O.S., Mitchell J.R. (2017). Biomedical Texture Analysis: Fundamentals, Tools and Challenges.

[36] Bębas E., Borowska M., Derlatka M., Oczeretko E., Hładuński M., Szumowski P., Mojsak M. (2021). Machine-learning-based classification of the histological subtype of non-small-cell lung cancer using mri texture analysis. Biomed. Signal Process. Control.

[37] Sohail A.S.M., Bhattacharya P., Mudur S.P., Krishnamurthy S. Local relative GLRLM-based texture feature extraction for classifying ultrasound medical images. Proceedings of the 24th Canadian Conference on Electrical and Computer Engineering (CCECE).

[38] Domino M., Romaszewski M., Jasiński T., Maśko M. (2020). Comparison of the surface thermal patterns of horses and donkeys in infrared thermography images. Animals.

[39] Masko M., Borowska M., Domino M., Jasinski T., Zdrojkowski L., Gajewski Z. (2021). A novel approach to thermographic images analysis of equine thoracolumbar region: The effect of effort and rider’s body weight on structural image complexity. BMC Vet. Res..

[40] Obuchowicz R., Nurzynska K., Obuchowicz B., Urbanik A., Piórkowski A. (2020). Caries detection enhancement using texture feature maps of intraoral radiographs. Oral Radiol..

[41] Pociask E., Nurzynska K., Obuchowicz R., Bałon P., Uryga D., Strzelecki M., Izworski A., Piórkowski A. (2021). Differential Diagnosis of Cysts and Granulomas Supported by Texture Analysis of Intraoral Radiographs. Sensors.

[42] Zhang H., Hung C.L., Min G., Guo J.P., Liu M., Hu X. (2019). Gpu-accelerated GLRLM algorithm for feature extraction of MRI. Sci. Rep..

[43] Martin B.B., Klide A.M. (1999). Physical examination of horses with back pain. Vet. Clin. N. Am..

[44] Davidson E.J. (2018). Lameness evaluation of the athletic horse. Vet. Clin. Equine Pract..

[45] Munsters C., van den Broek J., Welling E., van Weeren R., van Oldruitenborgh-Oosterbaan M.S. (2013). A prospective study on a cohort of horses and ponies selected for participation in the european eventing championship: Reasons for withdrawal and predictive value of fitness tests. BMC Vet. Res..

[46] Greve L., Dyson S. (2015). Saddle fit and management: An investigation of the association with equine thoracolumbar asymmetries, horse and rider health. Equine Vet. J..

[47] Szczypiński P.M., Klepaczko A., Depeursinge A., Al-Kadi O.S., Ross Mitchell J. (2017). MaZda—A framework for biomedical image texture analysis and data exploration. Biomedical Texture Analysis: Fundamentals, Tools and Challenges.

[48] Szczypiński P.M., Klepaczko A., Kociołek M. QmaZda—Software tools for image analysis and pattern recognition. Proceedings of the 2017 Signal Processing: Algorithms, Architectures, Arrangements, and Applications (SPA).

[49] Materka A., Strzelecki M. (1998). Texture Analysis Methods—A Review.

[50] Jain A.K. (1989). Fundamentals of Digital Image Processing.

[51] Daugman J.G. (1988). Complete discrete 2-d gabor transforms by neural networks for image analysis and compression. IEEE Trans. Signal Process..

[52] Lowe D.G. (2004). Distinctive image features from scale-invariant keypoints. Int. J. Comput. Vis..

[53] Haralick R.M. (1985). Statistical and structural approaches to texture. Digit. Image Process. Anal..

[54] Haralick R.M. (1979). Statistical and structural approaches to texture. Proc. IEEE.

[55] Fonseca B.P.A., Alves A.L.G., Nicoletti J.L.M., Thomassian A., Hussni C.A., Mikail S. (2006). Thermography and ultrasonography in back pain diagnosis of equine athletes. J. Equine Vet. Sci..

[56] Domino M., Borowska M., Trojakowska A., Kozłowska N., Zdrojkowski Ł., Jasiński T., Smyth G., Maśko M. (2022). The Effect of Rider: Horse Bodyweight Ratio on the Superficial Body Temperature of Horse’s Thoracolumbar Region Evaluated by Advanced Thermal Image Processing. Animals.

[57] Tunley B., Henson F. (2004). Reliability and repeatability of thermographic examination and the normal thermographic image of the thoracolumbar region in the horse. Equine Vet. J..

[58] Domino M., Borowska M., Kozłowska N., Zdrojkowski Ł., Jasiński T., Smyth G., Maśko M. (2022). Advances in Thermal Image Analysis for the Detection of Pregnancy in Horses Using Infrared Thermography. Sensors.

[59] Mircean M., Giurgiu G., Mircean V., Zinveliu E. (2007). Serum cortisol variation of sport horses in relation with the level of training and effort intensity. Bull. USAMV-CN.

[60] Viru A., Viru M. (2004). Cortisol-essential adaptation hormone in exercise. Int. J. Sports Med..

[61] Witkowska-Piłaszewicz O., Pingwara R., Winnicka A. (2020). The Effect of Physical Training on Peripheral Blood Mononuclear Cell Ex Vivo Proliferation, Differentiation, Activity, and Reactive Oxygen Species Production in Racehorses. Antioxidants.

[62] Witkowska-Piłaszewicz O., Grzędzicka J., Seń J., Czopowicz M., Żmigrodzka M., Winnicka A., Cywińska A., Carter C. (2021). Stress response after race and endurance training sessions and competitions in Arabian horses. Prev. Vet. Med..

[63] Witkowska-Piłaszewicz O., Kaszak I., Żmigrodzka M., Winnicka A., Sacharczuk M., Szczepaniak J., Cywińska A. (2019). Equine atypical myopathy—A review. Anim. Sci. Pap..

[64] Cywinska A., Gorecka R., Szarska E., Witkowski L., Dziekan P., Schollenberger A. (2010). Serum amyloid A level as a potential indicator of the status of endurance horses. Equine Vet. J. Suppl..

[65] Maśko M., Domino M., Jasiński T., Witkowska-Piłaszewicz O. (2021). The Physical Activity-Dependent Hematological and Biochemical Changes in School Horses in Comparison to Blood Profiles in Endurance and Race Horses. Animals.

[66] Häyrynen T.A.H. (2019). Smart Phone Thermal Camera Accessory Device as a Mean to Asses Saddle Fit in Horses. Master’s Thesis.

[67] Pereira N., Valenzuela D., Mangelsdorff G., Kufeke M., Roa R. (2018). Detection of perforators for free flap planning using smartphone thermal imaging: A concordance study with computed tomographic angiography in 120 perforators. Plast. Reconstr. Surg..

[68] Van Doremalen R.F.M., Van Netten J.J., Van Baal J.G., Vollenbroek-Hutten M.M.R., van der Heijden F. (2019). Validation of low-cost smartphone-based thermal camera for diabetic foot assessment. Diabetes Res. Clin. Pract..

[69] Jaiswal A., Amjad Z., Jha S., Sahni N., Chirayil S.B., Nair R.C. (2021). Accurate Device Temperature Forecasting using Recurrent Neural Network for Smartphone Thermal Management. Proceedings of the 2021 International Joint Conference on Neural Networks (IJCNN).

[70] Hall C., Burton K., Maycock E., Wragg E. (2011). A preliminary study into the use of infrared thermography as a means of assessing the horse’s response to different training methods. J. Vet. Behav..

[71] Clayton H., Dyson S., Harris P., Bondi A. (2015). Horses, saddles and riders: Applying the science. Equine Vet. Educ..

[72] Waran N., Randle H. (2017). What we can measure, we can manage: The importance of using robust welfare indicators in Equitation Science. Appl. Anim. Behav. Sci..

[73] Hall C., Randle H., Pearson G., Preshaw L., Waran N. (2018). Assessing equine emotional state. Appl. Anim. Behav. Sci..

[74] Randle H., Henshall C., Hall C., Pearson G., Preshaw L., Waran N. Indicators on the inside: Physiology and equine quality of life. Proceedings of the 15th International Conference of the International Society for Equitation Science.

[75] Redaelli V., Luzi F., Mazzola S., Bariffi G.D., Zappaterra M., Nanni Costa L., Padalino B. (2019). The use of infrared thermography (IRT) as stress indicator in horses trained for endurance: A pilot study. Animals.

[76] De Mira M.C., Lamy E., Santos R., Williams J., Pinto M.V., Martins P.S., Rodrigues P., Marlin D. (2021). Salivary cortisol and eye temperature changes during endurance competitions. BMC Vet. Res..

[77] Ibraheem N.A., Hasan M.M., Khan R.Z., Mishra P.K. (2012). Understanding color models: A review. JST.

[78] Wen C.-Y., Chou C.-M. (2004). Color image models and its applications to document examination. Forensic Sci. J..

[79] Maśko M., Zdrojkowski Ł., Wierzbicka M., Domino M. (2021). Association between the area of the highest flank temperature and concentrations of reproductive hormones during pregnancy in polish konik horses—A preliminary study. Animals.

[80] Plataniotis K.N., Venetsanopoulos A.N. (2013). Color Image Processing and Applications.

[81] Wilk I., Wnuk-Pawlak E., Janczarek I., Kaczmarek B., Dybczyńska M., Przetacznik M. (2020). Distribution of superficial body temperature in horses ridden by two riders with varied body weights. Animals.

[82] Mota-Rojas D., Pereira A.M., Wang D., Martínez-Burnes J., Ghezzi M., Hernández-Avalos I., Geraldo A.D.M. (2021). Clinical applications and factors involved in validating thermal windows used in infrared thermography in cattle and river buffalo to assess health and productivity. Animals.

[83] Soroko M., Howell K., Dudek K. (2017). The effect of ambient temperature on infrared thermographic images of joints in the distal forelimbs of healthy racehorses. J. Therm. Biol..

[84] Hodgson D., Davis R., McConaghy F. (1994). Thermoregulation in the horse in response to exercise. Br. Vet. J..

[85] Stubbs G. (2012). The Anatomy of the Horse.

[86] Von Schweinitz D.G. (1999). Thermographic diagnostics in equine back pain. Vet. Clin. N. Am. Equine Pract..

