# Supplementary material for: Selection of Image Texture Analysis and Color Model in the Advanced Image Processing of Thermal Images of Horses following Exercise
Source: Animals (Basel). 2022 Feb 12;12(4):444. doi: 10.3390/ani12040444 (PMC8868218; doi:10.3390/ani12040444)
Supplement: Supplementary file 1 [file animals-12-00444-s001.zip › animals-1519396-supplementary.pdf]

Supplementary Data for

Selection of image texture analysis and color model in the ad-vanced image processing of thermal images of the horse caused by exercise-induced fatigue

Małgorzata Domino [1], Marta Borowska [2], Natalia Kozłowska [1], Anna Trojakowska [3], Łukasz Zdrojkowski [1,*], Tomasz Jasiński [1] Graham Smyth[4], and Małgorzata Maśko [5,*]

[1] Department of Large Animal Diseases and Clinic, Institute of Veterinary Medicine, Warsaw University of Life Sciences (WULS–SGGW), Warsaw, 02–787, Poland; malgorzata_domino@sggw.edu.pl (M.D.); natalia_kozlowska@sggw.edu.pl (N.K.); tomasz_jasinski@sggw.edu.pl (T.J.)

[2] Institute of Biomedical Engineering, Faculty of Mechanical Engineering, Białystok University of Technology, Bialystok, 15-351, Poland; m.borowska@pb.edu.pl (M.B.)

[3] The Scientific Society of Veterinary Medicine Students, Warsaw University of Life Sciences, Warsaw, 02-787, Poland; an.trojakowska@gmail.com (A.T.)

[4] Menzies Health Institute Queensland and Griffith University School of Medicine, Southport, QLD, 4222, Australia; grahamcsmyth@gmail.com (G.S.)

[5] Department of Animal Breeding, Institute of Animal Science, Warsaw University of Life Sciences (WULS–SGGW), Warsaw, 02–787, Poland

* Correspondence: lukasz_zdrojkowski@sggw.edu.pl (Ł.Z.); malgorzata_masko@sggw.edu.pl (M.M.)

Table S1: Blood biomarkers level (mean±SD) before (BS 0) and after (BS 1) exercise in the model of six repetitions of exercise (REs).

**Blood biomarkers / exercise / *p***	**RE 1**	**RE 2**	**RE 3**	**RE 4**	**RE 5**	**RE 6**	***p***
**WBC, x10^9/L**							
BS 0	7.9±1.50^{a}	7.6±1.07^{a}	7.9±1.00^{a}	8.5±1.21^{a*}	7.9±1.68^{a*}	7.8±1.29^{a*}	0.742
BS 1	8.0±1.28^{a}	8.5±1.92^{ab}	8.7±1.27^{ab}	9.9±1.70^{ab*}	9.9±1.76^{ab*}	10.2±1.76^{b*}	**0.006**
p	0.758	0.202	0.127	**0.032**	**0.011**	**0.001**	
**RBC, x10^{12}/L**							
BS 0	7.6±0.56^{a*}	7.9±0.79^{a*}	7.9±0.77^{a*}	8.1±0.64^{a*}	7.7±0.71^{a*}	7.9±0.78^{a*}	0.802
BS 1	9.5±0.81^{a*}	9.4±0.86^{a*}	9.2±1.03^{a*}	9.8±1.41^{a*}	8.9±0.97^{a*}	9.1±0.84^{a*}	0.800
p	**0.0001**	**0.0001**	**0.0005**	**0.0001**	**0.001**	**0.0001**	
**HGB, mmol/L**							
BS 0	115±9.7^{a*}	119±9.4^{a*}	120±10.6^{a*}	123±11.2^{a*}	116±12.4^{a*}	119±13.1^{a*}	0.418
BS 1	145±13.4^{a*}	143±10.6^{a*}	141±17.1^{a*}	150±25.2^{a*}	136±17.0^{a*}	139±13.4^{a*}	0.400
p	**0.0001**	**0.0001**	**0.0003**	**0.0002**	**0.0004**	**0.0006**	
**HCT, %**							
BS 0	36.6±3.19^{a*}	37.8±3.07^{a*}	37.8±3.63^{a*}	38.9±3.50^{a*}	37.0±4.10^{a*}	37.8±3.71^{a*}	0.712
BS 1	46.1±3.87^{a*}	45.9±3.40^{a*}	44.9±5.22^{a*}	47.7±7.79^{a*}	43.9±5.35^{a*}	44.4±4.07^{a*}	0.690
p	**0.0001**	**0.0001**	**0.0002**	**0.0002**	**0.0003**	**0.0002**	
**MCV, fL**							
BS 0	48.1±2.44^{a}	48.3±2.34^{a}	48.2±2.21^{a}	48.5±2.38^{a}	48.3±2.61^{a}	48.2±2.36^{a}	0.118
BS 1	48.8±2.17^{a}	48.9±3.07^{a}	48.7±2.37^{a}	48.9±2.36^{a}	48.9±2.59^{a}	48.7±2.41^{a}	0.290
p	0.802	0.868	0.913	0.904	0.515	0.225	

**MCH, gL**							
BS 0	15.1±0.62^{a}	15.1±1.00^{a}	15.2±0.49^{a}	15.2±0.71^{a}	15.1±0.75^{a}	15.2±0.79^{a}	0.058
BS 1	15.3±0.61^{a}	15.3±0.92^{a}	15.2±0.62^{a}	15.4±0.76^{a}	15.2±0.69^{a}	14.4±2.79^{a}	0.072
p	0.0644	0.0780	0.5521	0.1840	0.2051	0.3430	
**MCHC, mmol/L**							
BS 0	315±5.4^{a}	315±8.7^{a}	316±7.7^{a}	315±6.2^{a}	313±5.9^{a}	316±8.8^{a}	0.647
BS 1	314±7.1^{a}	313±7.2^{a}	313±6.1^{a}	315±5.1^{a}	311±5.2^{a}	313±5.0^{a}	0.869
p	0.059	0.055	0.098	0.277	0.114	0.400	
**LAC, mmol/L**							
BS 0	0.1±0.02^{a*}	0.1±0.02^{a*}	0.1±0.01^{a*}	0.1±0.02^{a*}	0.1±0.03^{a*}	0.1±0.01^{a*}	0.858
BS 1	4.6±0.51^{a*}	4.7±0.24^{a*}	4.8±0.63^{a*}	4.8±0.71^{a*}	4.9±0.20^{a*}	4.9±0.34^{a*}	0.478
p	**<0.0001**	**<0.0001**	**<0.0001**	**<0.0001**	**<0.0001**	**<0.0001**	
**TSP, g/L**							
BS 0	5.90±0.38^{a}	6.29±0.63^{a}	5.96±0.43^{a}	6.02±0.33^{a}	6.37±0.88^{a}	6.15±0.47^{a}	0.858
BS 1	6.18±0.45^{a}	6.45±0.49^{a}	6.38±0.83^{a}	6.46±0.58^{a}	6.73±0.90^{a}	6.38±0.59^{a}	0.478
p	0.120	0.589	0.464	0.147	0.532	0.393	
**CPK, U/L**							
BS 0	157±58.3^{a}	148±40.2^{a*}	154±54.5^{a*}	176±30.8^{a*}	134±30.9^{a*}	148±45.3^{a*}	0.406
BS 1	202±85.3^{a}	209±65.5^{ab*}	211±50.8^{ab*}	239±102.0^{ab*}	245±65.2^{ab*}	294±70.9^{b*}	**0.048**
p	0.126	**0.008**	**0.022**	**0.025**	**0.0001**	**<0.0001**	
**ALT, U/L**							
BS 0	3.46±0.34^{a}	3.45±0.21^{a}	3.41±0.30^{a}	3.58±0.21^{a}	3.44±0.20^{a}	3.50±0.19^{a}	0.726
BS 1	3.61±0.34^{a}	3.57±0.28^{a}	3.52±0.22^{a}	3.55±0.22^{a}	3.55±0.41^{a}	3.48±0.22^{a}	0.295
p	0.236	0.427	0.063	0.538	0.284	0.148	
**AST, U/L**							
BS 0	274±59.8^{a}	274±42.3^{a}	280±54.5^{a}	274±46.8^{a}	263±19.1^{a*}	244±43.9^{a*}	0.323
BS 1	238±47.3^{a}	272±57.9^{ab}	280±39.9^{ab}	296±61.6^{ab}	282±33.8^{ab*}	309±51.8^{b*}	**0.022**
p	0.062	0.962	0.946	0.287	**0.049**	**0.018**	

WBC - white blood cell count; RBC - red blood cell count; HGB - hemoglobin concentration; HCT - hematocrit; MCV - mean corpuscular volume, MCH - mean corpuscular hemoglobin; MCHC - mean corpuscular hemoglobin concentration; LAC - blood lactate concentrations; TSP - total serum protein concentration; CPK - creatine phosphokinase activity; ALT - alanine aminotransferase activity; AST - aspartate aminotransferase activity. Asterisks and bolded *p*-value indicate differences between REs for $p < 0.05$. Lower case letters and bolded *p*-value indicate differences between BSs for $p < 0.05$.

Table S2: Body surface temperature (mean±SD) in four regions of interest (ROIs 1-4) before (BS 0) and after (BS 1) exercise in the model of six repetitions of exercise (REs).

**Surface temperature / exercise / *p***	**RE 1**	**RE 2**	**RE 3**	**RE 4**	**RE 5**	**RE 6**	***p***
**Tmax, ROI 1**							
BS 0	29.1±2.27^{a*}	31.6±2.93^{a*}	31.5±2.44^{a*}	29.1±2.28^{a*}	31.2±3.60^{a*}	31.6±2.39^{a*}	0.050
BS 1	39.1±1.59^{a*}	38.9±2.08^{a*}	39.0±1.99^{a*}	38.6±1.99^{a*}	38.5±2.69^{a*}	38.2±2.25^{a*}	0.908
p	**<0.0001**	**<0.0001**	**<0.0001**	**<0.0001**	**<0.0001**	**<0.0001**	
**Tmax, ROI 2**							
BS 0	29.9±3.30^{a*}	33.1±3.19^{a*}	32.3±2.90^{a*}	30.8±1.66^{a*}	31.8±2.40^{a*}	31.5±2.55^{a*}	0.068
BS 1	38.3±1.37^{a*}	38.6±1.91^{a*}	38.9±2.20^{a*}	37.8±3.12^{a*}	38.2±3.16^{a*}	38.6±2.42^{a*}	0.904
p	**<0.0001**	**<0.0001**	**<0.0001**	**<0.0001**	**<0.0001**	**<0.0001**	
**Tmax, ROI 3**							
BS 0	29.5±3.31^{a*}	32.2±3.77^{a*}	32.2±2.98^{a*}	29.9±2.27^{a*}	31.1±3.01^{a*}	31.5±2.57^{a*}	0.050
BS 1	38.7±1.65^{a*}	39.2±2.07^{a*}	39.2±2.07^{a*}	38.5±2.40^{a*}	38.8±2.88^{a*}	39.1±1.95^{a*}	0.968
p	**<0.0001**	**<0.0001**	**<0.0001**	**<0.0001**	**<0.0001**	**<0.0001**	
**Tmax, ROI 4**							
BS 0	29.9±2.98^{a*}	33.0±2.90^{a*}	32.4±2.72^{a*}	30.4±2.58^{a*}	31.6±3.15^{a*}	31.6±2.64^{a*}	0.065
BS 1	39.0±1.76^{a*}	39.2±2.04^{a*}	37.0±2.20^{a*}	38.6±2.56^{a*}	38.5±2.88^{a*}	39.5±2.02^{a*}	0.123
p	**<0.0001**	**<0.0001**	**<0.0001**	**<0.0001**	**<0.0001**	**<0.0001**	
**Taver, ROI 1**							
BS 0	25.5±4.05^{a*}	28.1±3.89^{a*}	27.8±3.39^{a*}	25.2±2.09^{a*}	25.8±2.21^{a*}	27.5±1.57^{a*}	0.065
BS 1	35.8±2.35^{a*}	36.9±2.14^{a*}	37.0±2.17^{a*}	35.3±2.40^{a*}	35.7±2.54^{a*}	36.6±1.68^{a*}	0.502
p	**<0.0001**	**<0.0001**	**<0.0001**	**<0.0001**	**<0.0001**	**<0.0001**	
**Taver, ROI 2**							
BS 0	27.2±2.83^{a*}	29.9±3.67^{a*}	29.7±3.07^{a*}	28.1±1.97^{a*}	28.6±1.79^{a*}	29.9±1.41^{a*}	0.055
BS 1	35.3±1.76^{a*}	36.0±1.90^{a*}	36.5±2.07^{a*}	34.9±2.64^{a*}	35.1±2.93^{a*}	36.1±2.26^{a*}	0.417
p	**<0.0001**	**<0.0001**	**<0.0001**	**<0.0001**	**<0.0001**	**<0.0001**	
**Taver, ROI 3**							
BS 0	25.1±3.16^{a*}	27.3±3.15^{a*}	27.6±3.15^{a*}	24.6±2.12^{a*}	25.3±1.96^{a*}	26.4±2.48^{a*}	0.070
BS 1	35.2±2.35^{a*}	36.4±2.26^{a*}	36.9±2.38^{a*}	34.8±2.55^{a*}	35.1±2.84^{a*}	35.9±2.21^{a*}	0.240
p	**<0.0001**	**<0.0001**	**<0.0001**	**<0.0001**	**<0.0001**	**<0.0001**	
**Taver, ROI 4**							
BS 0	25.7±3.62^{a*}	27.3±3.83^{a*}	27.6±2.86^{a*}	25.0±2.33^{a*}	25.5±2.21^{a*}	27.0±2.02^{a*}	0.079
BS 1	35.3±2.55^{a*}	36.6±2.29^{a*}	37.7±1.56^{a*}	34.9±2.69^{a*}	35.1±2.77^{a*}	36.3±2.15^{a*}	0.059
p	**<0.0001**	**<0.0001**	**<0.0001**	**<0.0001**	**<0.0001**	**<0.0001**	

Tmax - maximal temperature; Taver - average temperature. Asterisks and bolded *p*-value indicate differences between REs for $p < 0.05$. Lower case letters and bolded *p*-value indicate differences between BSs for $p < 0.05$.

Table S3: Selected features of image texture analysis (mean±SD) in four regions of interest (ROIs 1-4) before (BS 0) and after (BS 1) exercise in the model of six repetitions of exercise (REs).

**Structural image complexity / exercise / *p***	**RE 1**	**RE 2**	**RE 3**	**RE 4**	**RE 5**	**RE 6**	***p***
**R.HS.Variance, ROI 1**							
BS 0	68±26.4^{a*}	47±14.1^{a*}	51±30.7^{a*}	66±29.7^{a*}	73±33.3^{a*}	66±37.7^{a*}	0.230
BS 1	5±1.4^{a*}	7±2.0^{a*}	5±1.5^{a*}	31±8.9^{b*}	22±6.4^{b*}	41±12.0^{b*}	**<0.0001**
p	**<0.0001**	**<0.0001**	**<0.0001**	**0.033**	**0.0004**	**0.010**	
**R.HS.Variance, ROI 2**							
BS 0	13±16.2^{ab}	3±6.8^{a*}	4±4.3^{a*}	33±34.5^{b*}	34±30.7^{b*}	15±25.3^{ab*}	**<0.0001**
BS 1	2±4.2^{a}	5±1.4^{ac*}	3±2.9^{ac*}	17±4.9^{b*}	13±3.7^{bc*}	9±13.9^{ab*}	**0.0002**
p	0.068	0.404	0.506	0.314	0.051	0.458	
**R.HS.Variance, ROI 3**							
BS 0	108±40.7^{a*}	84±47.4^{a*}	75±40.0^{a*}	104±38.2^{a*}	98±31.3^{a*}	93±47.6^{a*}	0.336
BS 1	6±6.5^{ab*}	14±17.1^{ab*}	2±1.6^{a*}	31±29.8^{b*}	19±14.4^{b*}	26±36.1^{b*}	**<0.0001**
p	**<0.0001**	**<0.0001**	**<0.0001**	**<0.0001**	**<0.0001**	**<0.0001**	
**R.HS.Variance, ROI 4**							
BS 0	92±28.3^{a*}	91±49.6^{a*}	71±31.0^{a*}	101±32.5^{a*}	106±33.8^{a*}	101±36.6^{a*}	0.154
BS 1	10±11.1^{ab*}	11±12.9^{ab*}	4±3.2^{a*}	31±28.8^{b*}	19±12.9^{b*}	22±23.3^{b*}	**0.0001**
p	**<0.0001**	**<0.0001**	**<0.0001**	**<0.0001**	**<0.0001**	**<0.0001**	
**R.GLCM.SumOfSqs, ROI 1**							
BS 0	64±32.2^{ab*}	30±23.7^{a*}	28±25.9^{a*}	83±40.7^{b*}	80±41.6^{b*}	52±36.8^{ab*}	**0.0005**
BS 1	5±4.8^{a*}	9±6.8^{ac*}	5±5.0^{a*}	40±40.0^{b*}	26±22.0^{bc*}	31±41.4^{bc*}	**<0.0001**
p	**<0.0001**	**0.010**	**0.010**	**0.010**	**0.001**	**0.028**	
**R.GLCM.SumOfSqs, ROI 2**							
BS 0	12±16.1^{ab}	3±6.8^{a}	4±4.2^{a}	33±34.4^{b}	34±30.6^{b*}	15±25.1^{ab}	**<0.0001**
BS 1	2±4.2^{a}	4±4.9^{ab}	2±2.9^{a}	18±17.1^{b}	11±12.8^{b*}	9±13.8^{ab}	**0.0002**
p	0.068	0.404	0.506	0.314	**0.033**	0.701	
**R.GLCM.SumOfSqs, ROI 3**							
BS 0	125±39.7^{a*}	114±83.5^{ab*}	69±47.0^{b*}	104±38.2^{ab*}	114±30.0^{ab*}	92±47.5^{ab*}	**0.031**
BS 1	6±6.5^{ab*}	14±17.1^{a*}	1±1.5^{b*}	31±29.7^{a*}	19±14.3^{a*}	26±36.0^{a*}	**<0.0001**
p	**<0.0001**	**<0.0001**	**<0.0001**	**<0.0001**	**<0.0001**	**<0.0001**	
**R.GLCM.SumOfSqs, ROI 4**							
BS 0	116±27.3^{ab*}	85±55.0^{ab*}	64±39.9^{a*}	117±30.1^{b*}	123±28.7^{b*}	101±36.6^{ab*}	**0.005**
BS 1	10±11.0^{ab*}	11±12.9^{ab*}	3±3.1^{a*}	31±28.7^{b*}	19±12.7^{b*}	26±36.0^{ab*}	**0.0008**
p	**<0.0001**	**<0.0001**	**<0.0001**	**<0.0001**	**<0.0001**	**<0.0001**	
**R.GLCM.SumVarnc, ROI 1**							
BS 0	257±128.5^{ab*}	120±94.6^{a*}	111±103.6^{a*}	329±162.7^{b*}	318±166.2^{b*}	207±147.0^{ab*}	**0.005**
BS 1	16±19.1^{a*}	34±27.3^{ac*}	18±19.9^{a*}	161±123.7^{b*}	102±97.9^{bc*}	125±165.3^{bc*}	**<0.0001**
p	**<0.0001**	**0.010**	**0.010**	**0.010**	**0.001**	**0.028**	
**R.GLCM.SumVarnc, ROI 2**							
BS 0	49±61.7^{ab}	11±26.1^{a}	14±15.8^{a}	132±131.4^{b}	134±117.2^{b*}	60±96.0^{ab}	**<0.0001**
BS 1	8±16.9^{a}	14±19.7^{ab}	8±11.6^{a}	71±68.0^{b}	44±50.1^{b*}	35±55.1^{ab}	**0.0002**

p	0.051	0.703	0.360	0.189	**0.033**	**0.463**	
**R.GLCM.SumVarnc, ROI 3**							
BS 0	499±152.1[a*]	334±181.3[ab*]	276±179.7[b*]	415±146.1[ab*]	457±114.8[ab*]	369±181.9[ab*]	**0.031**
BS 1	25±25.9[ab*]	57±68.3[a*]	6±6.2[b*]	124±118.6[a*]	76±57.3[a*]	102±143.9[a*]	**<0.0001**
p	**<0.0001**	**<0.0001**	**<0.0001**	**<0.0001**	**<0.0001**	**<0.0001**	
**R.GLCM.SumVarnc, ROI 4**							
BS 0	465±109.9[ab*]	339±219.7[ab*]	254±159.2[a*]	467±120.3[b*]	489±114.8[b*]	401±146.2[ab*]	**0.044**
BS 1	41±43.9[a*]	43±51.5[a*]	13±12.6[a*]	123±114.7[a*]	74±50.9[a*]	75±97.9[a*]	**0.0006**
p	**<0.0001**	**<0.0001**	**<0.0001**	**<0.0001**	**<0.0001**	**<0.0001**	
**r.HS.Variance, ROI 1**							
BS 0	90±48.0[a*]	58±47.2[a*]	54±36.9[a*]	99±43.7[a*]	92±47.2[a*]	62±49.7[a]	0.055
BS 1	14±6.1[a*]	14±5.8[a*]	16±6.0[a*]	49±46.9[b*]	35±44.1[ab*]	42±49.7[ab]	**0.013**
p	**0.0002**	**0.008**	**0.004**	**0.014**	**0.005**	0.323	
**r.HS.Variance, ROI 2**							
BS 0	26±14.6[a*]	17±3.9[a*]	17±4.2[a*]	31±20.2[a*]	35±22.0[a*]	26±31.5[a*]	0.120
BS 1	21±5.2[a*]	18±6.6[a*]	20±5.9[a*]	27±18.2[a*]	21±9.9[a*]	23±13.1[a*]	0.867
p	0.334	0.473	0.182	0.631	0.076	0.736	
**r.HS.Variance, ROI 3**							
BS 0	107±27.3[a*]	96±49.1[a*]	85±46.7[a*]	115±31.2[a*]	123±19.2[a*]	93±42.0[a*]	0.146
BS 1	18±4.2[a*]	21±15.9[a*]	16±4.9[a*]	45±49.0[a*]	23±13.0[a*]	33±27.9[a*]	0.343
p	**<0.0001**	**0.0002**	**0.0003**	**0.0005**	**<0.0001**	**0.0005**	
**r.HS.Variance, ROI 4**							
BS 0	128±29.4[a*]	93±50.5[a*]	91±53.6[a*]	128±15.9[a*]	123±14.4[a*]	111±36.4[a*]	0.483
BS 1	17±6.6[a*]	18±9.5[a*]	13±3.9[a*]	52±62.7[a*]	19±10.1[a*]	28±39.5[a*]	0.162
p	**<0.0001**	**0.0003**	**0.0004**	**0.002**	**<0.0001**	**<0.0001**	
**r.GLCM.SumOfSqs, ROI 1**							
BS 0	89±48.1[a*]	58±42.9[a*]	54±33.4[a*]	99±43.8[a*]	84±46.0[a*]	62±49.7[a]	0.074
BS 1	14±6.2[a*]	14±5.8[a*]	16±6.0[a*]	49±46.9[b*]	35±44.2[ab*]	42±49.7[b]	**0.014**
p	**0.0002**	**0.004**	**0.002**	**0.014**	**0.014**	0.322	
**r.GLCM.SumOfSqs, ROI 2**							
BS 0	26±14.6[a]	17±3.9[a]	17±4.3[a]	31±20.1[a]	35±22.0[a]	26±31.6[a]	0.122
BS 1	21±5.3[a]	18±6.6[a]	20±6.0[a]	27±18.9[a]	21±9.9[a]	23±13.0[a]	0.866
p	0.361	0.432	0.151	0.651	0.081	0.474	
**r.GLCM.SumOfSqs, ROI 3**							
BS 0	107±27.5[a*]	96±49.3[a*]	85±46.8[a*]	115±31.9[a*]	123±19.2[a*]	93±42.2[a*]	0.141
BS 1	18±4.2[a*]	21±15.9[a*]	16±4.9[a*]	45±49.0[a*]	22±12.9[a*]	33±27.9[a*]	0.351
p	**<0.0001**	**0.0002**	**0.0003**	**0.0005**	**<0.0001**	**0.0005**	
**r.GLCM.SumOfSqs, ROI 4**							
BS 0	128±29.4[a*]	93±50.7[a*]	91±53.8[a*]	128±15.8[a*]	123±14.3[a*]	111±36.5[a*]	0.509
BS 1	16±6.7[a*]	18±9.5[a*]	13±3.9[a*]	51±62.7[a*]	19±9.9[a*]	28±39.5[a*]	0.168
p	**<0.0001**	**0.0003**	**0.0004**	**0.001**	**<0.0001**	**<0.0001**	
**r.GLCM.SumVarnc, ROI 1**							
BS 0	349±185.1[ab*]	184±179.2[a*]	164±135.4[a*]	411±173.4[b*]	383±186.0[b*]	427±138.5[b*]	**0.0002**
BS 1	55±24.5[a*]	56±23.2[a*]	63±24.0[a*]	193±179.6[b*]	137±170.2[ab*]	160±188.3[ab*]	**0.015**
p	**0.0002**	**0.0308**	**0.026**	**0.006**	**0.003**	**0.0008**	

**r.GLCM.SumVarnc, ROI 2**							
BS 0	102±58.2^{a}	65±15.6^{a}	65±16.9^{a}	120±77.7^{a}	136±85.3^{a}	102±122.2^{a}	0.115
BS 1	85±20.8^{a}	72±26.6^{a}	78±23.8^{a}	107±72.2^{a}	85±39.6^{a}	90±51.5^{a}	0.871
p	0.353	0.441	0.152	0.673	0.079	0.761	
**r.GLCM.SumVarnc, ROI 3**							
BS 0	417±104.8^{a*}	370±187.7^{a*}	331±181.1^{a*}	446±120.0^{a*}	474±77.4^{a*}	362±161.2^{a*}	0.144
BS 1	71±16.9^{a*}	83±63.4^{a*}	64±19.4^{a*}	176±189.9^{a*}	89±51.7^{a*}	129±109.0^{a*}	0.344
p	**<0.0001**	**0.0002**	**0.0003**	**0.0006**	**<0.0001**	**0.0005**	
**r.GLCM.SumVarnc, ROI 4**							
BS 0	498±117.2^{a*}	362±195.4^{a*}	353±205.7^{a*}	494±64.1^{a*}	476±56.7^{a*}	427±138.5^{a*}	0.439
BS 1	66±26.7^{a*}	71±38.0^{a*}	53±15.6^{a*}	199±237.8^{a*}	76±39.8^{a*}	111±154.3^{a*}	0.165
p	**<0.0001**	**<0.0001**	**<0.0001**	**<0.0001**	**<0.0001**	**<0.0001**	
**r.GLCH.SumOfSqs, ROI 1**							
BS 0	89±48.1^{a*}	63±50.7^{a*}	58±43.5^{a*}	99±43.8^{a*}	101±49.7^{a*}	62±49.8^{a*}	0.085
BS 1	14±6.2^{a*}	14±5.8^{a*}	16±6.1^{a*}	49±46.9^{b*}	35±44.2^{ab*}	42±49.7^{ab*}	**0.013**
p	**0.0002**	**0.006**	**0.006**	**0.014**	**0.002**	0.323	
**r.GLCH.SumOfSqs, ROI 2**							
BS 0	26±14.7^{a}	16±3.9^{a}	16±4.3^{a}	30±20.1^{a}	34±22.0^{a}	26±31.7^{a*}	0.051
BS 1	21±5.3^{a}	18±6.7^{a}	20±6.0^{a}	27±18.2^{a}	21±10.0^{a}	23±13.0^{a*}	0.876
p	0.366	0.407	0.149	0.674	0.083	0.764	
**r.GLCH.SumOfSqs, ROI 3**							
BS 0	107±27.4^{a*}	96±49.1^{a*}	85±46.6^{a*}	115±31.8^{a*}	123±19.2^{a*}	94±42.1^{a*}	0.145
BS 1	18±4.2^{a*}	21±15.9^{a*}	16±4.8^{a*}	45±49.0^{a*}	23±13.1^{a*}	33±28.0^{a*}	0.350
p	**<0.0001**	**0.0002**	**0.0003**	**0.0005**	**<0.0001**	**0.0005**	
**r.GLCH.SumOfSqs, ROI 4**							
BS 0	128±29.5^{a*}	96±49.1^{a*}	91±53.9^{a*}	115±31.8^{a*}	124±14.4^{a*}	111±36.7^{a*}	0.727
BS 1	16±6.7^{a*}	18±9.5^{a*}	13±4.0^{a*}	52±62.8^{a*}	19±9.8^{a*}	28±39.6^{a*}	0.168
p	**<0.0001**	**0.0002**	**0.0004**	**0.006**	**<0.0001**	**<0.0001**	
**r.GLCH.SumVarnc, ROI 1**							
BS 0	307±167.1^{a*}	234±180.1^{a*}	222±147.0^{a*}	411±173.4^{a*}	341±186.2^{a*}	239±186.8^{a}	0.081
BS 1	55±24.5^{a*}	56±23.2^{a*}	63±24.0^{a*}	193±179.6^{b*}	137±170.0^{ab*}	161±188.3^{ab}	**0.015**
p	**0.0003**	**0.006**	**0.003**	**0.006**	**0.010**	0.317	
**r.GLCH.SumVarnc, ROI 2**							
BS 0	102±58.2^{ab*}	65±15.6^{a*}	65±16.9^{a*}	121±77.7^{ab*}	136±85.3^{b*}	102±122.2^{ab*}	**0.008**
BS 1	85±20.8^{a*}	72±26.6^{a*}	78±23.8^{a*}	107±72.2^{a*}	85±39.6^{a*}	90±51.5^{a*}	0.871
p	0.353	0.441	0.152	0.673	0.079	0.761	
**r.GLCH.SumVarnc, ROI 3**							
BS 0	417±104.8^{a*}	370±187.7^{a*}	331±181.1^{a*}	446±120.0^{a*}	474±77.4^{a*}	362±161.2^{a*}	0.144
BS 1	71±16.9^{a*}	83±63.4^{a*}	64±19.4^{a*}	176±189.9^{a*}	89±51.7^{a*}	129±109.0^{a*}	0.344
p	**<0.0001**	**0.0002**	**0.0003**	**0.0006**	**<0.0001**	**0.0005**	
**r.GLCH.SumVarnc, ROI 4**							
BS 0	498±117.2^{a*}	362±195.4^{a*}	353±205.7^{a*}	494±64.1^{a*}	476±56.7^{a*}	427±138.5^{a*}	0.439
BS 1	66±26.7^{a*}	71±38.0^{a*}	53±15.6^{a*}	199±237.8^{a*}	76±39.8^{a*}	111±154.3^{a*}	0.165
p	**<0.0001**	**<0.0001**	**<0.0001**	**<0.0001**	**<0.0001**	**<0.0001**	

**B.GLCM.SumVarnc, ROI 1**							
BS 0	73±46.2^{ab*}	36±29.7^{ab}	20±20.5^{a}	146±135.4^{ab*}	126±108.2^{b*}	59±65.2^{b}	**0.001**
BS 1	29±8.2^{a*}	32±8.0^{ab}	34±11.2^{ab}	58±32.2^{b*}	46±22.1^{ab*}	62±61.6^{ab}	**0.005**
p	**0.007**	0.743	0.052	**0.049**	**0.026**	0.288	
**B.GLCM.SumVarnc, ROI 2**							
BS 0	22±7.9^{ab*}	24±7.6^{ab*}	21±6.1^{a}	50±52.9^{ab}	48±45.2^{b}	29±24.1^{ab}	**0.007**
BS 1	14±7.4^{a*}	15±7.2^{a*}	17±3.9^{a}	19±12.9^{a}	21±12.4^{a}	18±10.4^{a}	0.253
p	**0.004**	**0.017**	0.105	0.070	0.074	0.177	
**B.GLCM.SumVarnc, ROI 3**							
BS 0	325±161.6^{a*}	140±134.7^{ab*}	91±109.8^{b}	242±176.5^{ab*}	279±148.6^{a*}	171±149.2^{ab*}	**0.002**
BS 1	35±12.6^{a*}	40±8.7^{a*}	33±5.7^{a}	54±30.3^{a*}	41±10.7^{a*}	55±46.5^{a*}	0.082
p	**<0.0001**	**0.026**	0.743	**0.017**	**<0.0001**	**<0.0001**	
**B.GLCM.SumVarnc, ROI 4**							
BS 0	290±145.5^{a*}	153±137.8^{ab*}	78±73.8^{b*}	281±159.4^{a*}	316±146.0^{a*}	187±106.5^{ab*}	**0.0004**
BS 1	37±11.7^{ab*}	36±10.4^{ab*}	31±6.8^{a*}	50±22.4^{b*}	42±15.8^{ab*}	48±22.0^{b*}	**0.013**
p	**<0.0001**	**0.013**	**0.049**	**0.0004**	**<0.0001**	**<0.0001**	
**Q.HS.Variance, ROI 1**							
BS 0	724±370^{a*}	905±353^{a*}	713±410^{a*}	511±265^{a*}	537±309^{a*}	741±450^{a*}	0.113
BS 1	1411±305^{a*}	1590±400^{ab*}	1608±421^{ab*}	1640±339^{ab*}	1731±391^{b*}	1633±464^{ab*}	**0.018**
p	**<0.0001**	**<0.0001**	**<0.0001**	**<0.0001**	**<0.0001**	**<0.0001**	
**Q.HS.Variance, ROI 2**							
BS 0	1309±369^{a*}	1317±358^{a*}	1369±360^{a}	1476±306^{a*}	1473±521^{a*}	1398±490^{a*}	0.590
BS 1	751±213^{a*}	887±283^{ab*}	1052±199^{ab*}	874±303^{ab*}	989±351^{b*}	1064±287^{ab*}	**0.045**
p	**<0.0001**	**0.006**	**0.045**	**<0.0001**	**0.015**	**0.046**	
**Q.HS.Variance, ROI 3**							
BS 0	848±245^{a*}	907±273^{a*}	754±269^{a*}	674±256^{a*}	700±274^{a*}	719±317^{a*}	0.234
BS 1	1614±509^{ab*}	1940±264^{a*}	1407±295^{b*}	1823±236^{ab*}	1857±320^{ab*}	1691±475^{ab*}	**0.009**
p	**0.0002**	**<0.0001**	**<0.0001**	**<0.0001**	**<0.0001**	**<0.0001**	
**Q.HS.Variance, ROI 4**							
BS 0	797±192^{a*}	896±375^{a*}	698±357^{a*}	718±303^{a*}	738±292^{a*}	537±264^{a*}	0.171
BS 1	1676±290^{ab*}	1670±413^{ab*}	1435±332^{b*}	1824±304^{a*}	1797±317^{ab*}	1807±367^{a*}	**0.040**
p	**<0.0001**	**<0.0001**	**<0.0001**	**<0.0001**	**<0.0001**	**<0.0001**	
**H.HS.Perc99, ROI 1**							
BS 0	204±31.3^{a*}	176±42.6^{ab*}	155±29.4^{b*}	213±21.5^{b*}	209±31.3^{b*}	186±30.0^{ab}	**0.0006**
BS 1	131±25.5^{a*}	137±18.4^{a*}	127±10.3^{a*}	186±34.1^{b*}	168±33.8^{b*}	162±38.6^{b}	**<0.0001**
p	**<0.0001**	**0.029**	**0.003**	**0.015**	**0.005**	0.058	
**H.HS.Perc99, ROI 2**							
BS 0	140±30.3^{ab}	124±18.3^{a}	125±8.9^{ab}	173±40.3^{b}	178±36.9^{b*}	142±31.1^{ab}	**<0.0001**
BS 1	118±8.1^{a}	123±7.8^{a}	121±6.9^{a}	148±27.1^{b}	142±31.4^{b*}	135±24.2^{b}	**<0.0001**
p	0.080	0.467	0.325	0.183	**0.010**	0.540	
**H.HS.Perc99, ROI 3**							
BS 0	225±12.0^{a*}	205±27.9^{ab*}	189±34.9^{b*}	218±15.4^{ab*}	226±5.5^{a*}	205±30.6^{ab*}	**0.0005**
BS 1	131±18.3^{ab*}	142±27.2^{ab*}	120±6.5^{b*}	169±39.1^{b*}	157±34.0^{b*}	152±41.5^{b*}	**<0.0001**
p	**<0.0001**	**<0.0001**	**<0.0001**	**0.001**	**<0.0001**	**0.002**	
**H.HS.Perc99, ROI 4**							

BS 0	226±4.4^{a*}	197±38.6^{ab*}	190±35.7^{b*}	222±9.5^{a*}	224±8.3^{a*}	213±26.6^{ab*}	**0.0003**
BS 1	144±32.0^{ab*}	141±29.2^{ab*}	125±9.1^{b*}	165±32.9^{b*}	158±28.1^{b*}	156±37.7^{b*}	**0.002**
p	**<0.0001**	**0.008**	**<0.0001**	**<0.0001**	**<0.0001**	**0.004**	
**B.HS.Perc99, ROI 1**							
BS 0	160±60.0^{ab*}	112±69.2^{ab}	74±38.4^{a}	178±52.9^{b*}	172±52.9^{b*}	120±62.7^{ab}	**0.002**
BS 1	87±25.6^{a*}	84±11.2^{a}	87±9.1^{a}	133±50.3^{b*}	113±43.6^{ab*}	113±54.7^{ab}	**0.018**
p	**0.002**	0.175	0.294	**0.043**	**0.018**	0.787	
**B.HS.Perc99, ROI 2**							
BS 0	75±17.4^{a}	74±9.9^{a}	71±7.3^{a}	113±58.6^{a}	117±56.1^{a}	83±40.5^{a}	0.504
BS 1	71±12.8^{a}	69±14.4^{a}	70±10.3^{a}	74±32.3^{a}	82±35.8^{a}	70±16.1^{a}	0.755
p	0.590	0.310	0.751	0.062	0.082	0.292	
**B.HS.Perc99, ROI 3**							
BS 0	204±34.5^{a*}	163±59.4^{ab*}	133±63.8^{b*}	189±44.2^{ab*}	212±11.1^{a*}	160±69.4^{ab*}	**0.003**
BS 1	84±8.4^{a*}	93±20.3^{a*}	89±8.8^{a*}	122±53.3^{a*}	104±30.2^{a*}	112±51.8^{a*}	0.372
p	**<0.0001**	**0.002**	**0.035**	**0.003**	**<0.0001**	**0.043**	
**B.HS.Perc99, ROI 4**							
BS 0	214±8.4^{a*}	157±70.4^{ab*}	136±63.5^{b*}	200±26.4^{a*}	207±24.0^{a*}	182±62.9^{ab*}	**0.010**
BS 1	99±33.2^{a*}	92±23.6^{a*}	84±9.5^{a*}	110±44.7^{a*}	99±35.7^{a*}	110±38.8^{a*}	0.399
p	**<0.0001**	**0.009**	**0.017**	**<0.0001**	**<0.0001**	**0.003**	

R.HS.Variance - Variance of histogram statistics, Red component in RGB color model; R.GLCM.SumOfSqs - Sum of Squares of Grey-Level Co-occurrence Matrix, Red component in RGB color model; R.GLCM.SumVarnc - Sum of Variance of Grey-Level Co-occurrence Matrix, Red component in RGB color model; r.HS.Variance - Normalized Variance of histogram statistics, Red component in RGB color model; r.GLCM.SumOfSqs - Normalized Sum of Squares of Grey-Level Co-occurrence Matrix, Red component in RGB color model; r.GLCM.SumVarnc - Normalized Sum of Variance of Grey-Level Co-occurrence Matrix, Red component in RGB color model; r.GLCH.SumOfSqs - Normalized Sum of Squares of the asymmetric Gray Level Co-occurrence Matrix, Red component in RGB color model; r.GLCH.SumVarnc - Normalized Sum of Variance of the asymmetric Gray Level Co-occurrence Matrix, Red component in RGB color model; B.GLCM.SumVarnc - Sum of Variance of Grey-Level Co-occurrence Matrix, Blue component in RGB color model; Q.HS.Variance - Variance of histogram statistics, Q-component in YIQ color model; H.HS.Perc99 - Percentiles 99 of histogram statistics, Hue component in HSB color model; B.HS.Perc99 - Percentiles 99 of histogram statistics, Brightness component in HSB color model. Asterisks and bolded *p*-value indicate differences between REs for $p < 0.05$. Lower case letters and bolded *p*-value indicate differences between BSs for $p < 0.05$.
